# Establishing a Cell-Free Transcription–Translation Platform for *Cutibacterium acnes* to Prototype Engineered Metabolic and Synthetic Biology

**DOI:** 10.1021/acsbiomaterials.1c00894

**Published:** 2021-12-31

**Authors:** María-José Fábrega, Nastassia Knödlseder, Guillermo Nevot, Marta Sanvicente, Lorena Toloza, Javier Santos-Moreno, Marc Güell

**Affiliations:** Department of Experimental and Health Sciences, Pompeu Fabra University, Carrer del Dr. Aiguader 88, 00803 Barcelona, Spain

**Keywords:** cell-free system, *Cutibacterium acnes*, biosensor, firefly luciferase, FMN-GFP, RNA-seq

## Abstract

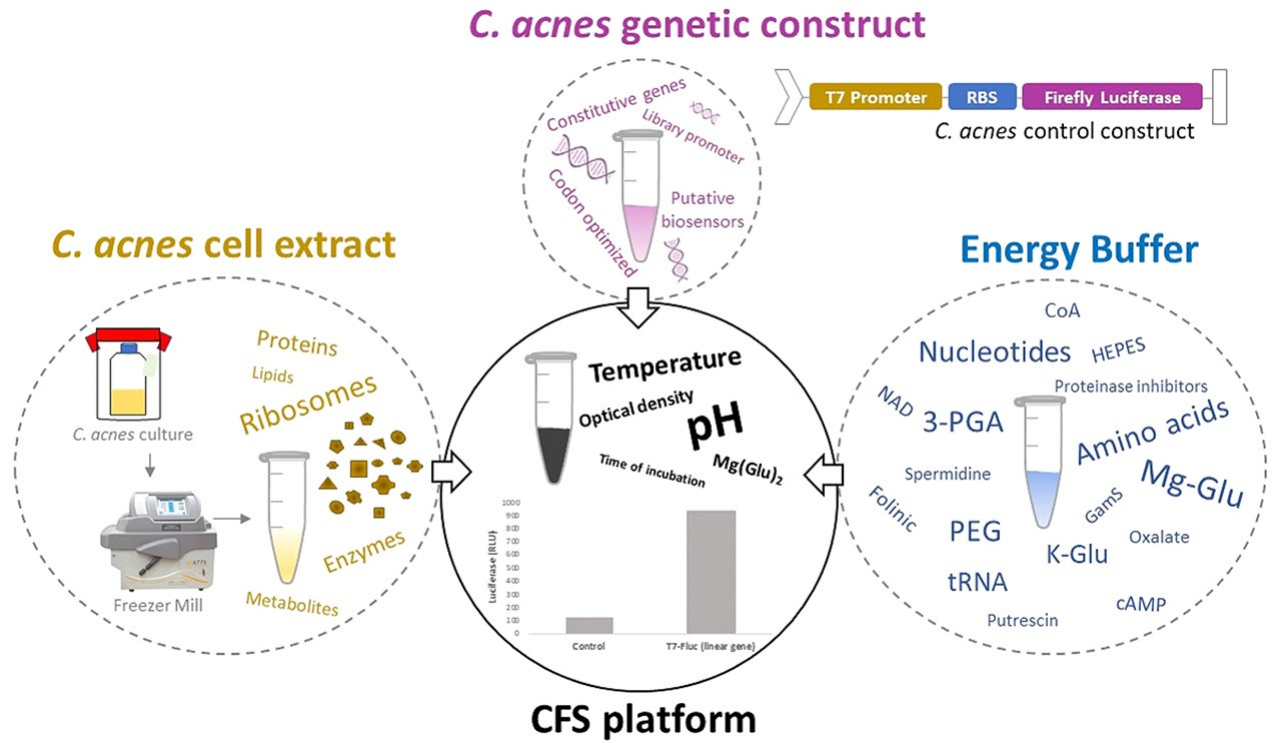

In the past few years, new bacterial-cell-free transcription–translation systems have emerged as potent and quick platforms for protein production as well as for prototyping of DNA regulatory elements, genetic circuits, and metabolic pathways. The Gram-positive commensal *Cutibacterium acnes* is one of the most abundant bacteria present in the human skin microbiome. However, it has recently been reported that some *C. acnes* phylotypes can be associated with common inflammatory skin conditions, such as acne vulgaris, whereas others seem to play a protective role, acting as possible “skin probiotics”. This fact has made *C. acnes* become a bacterial model of interest for the cosmetic industry. In the present study we report for the first time the development and optimization of a *C. acnes*-based cell-free system (CFS) that is able to produce 85 μg/mL firefly luciferase. We highlight the importance of harvesting the bacterial pellet in mid log phase and maintaining CFS reactions at 30 °C and physiological pH to obtain the optimal yield. Additionally, a *C. acnes* promoter library was engineered to compare coupled *in vitro* TX-TL activities, and a temperature biosensor was tested, demonstrating the wide range of applications of this toolkit in the synthetic biology field.

## Introduction

From the moment human beings are born, their skin lives in a continuous interaction with a highly diverse and complex microbial population called skin microbiota. The main function of this bacterial barrier is to establish a symbiosis with the host to maintain skin health.^1^ In general, this bacterial community changes its composition throughout the different stages of life, but when the human body reaches adulthood, the variability is reduced, and the skin microbiota tend to remain stable.^2^ Nevertheless, in most skin-related diseases, such as acne vulgaris, it has recently been demonstrated that the abundance and bacterial composition is altered, especially for *Cutibacterium acnes*, one of the most predominant bacteria in the skin microbiota. In this regard, it has been proven that specific phylogenetic types of *C. acnes* are responsible and directly involved in acne vulgaris while a few others are not.^3^ Therefore, it seems that this bacterial strain plays a critical role during particular skin alteration processes.

However, despite being a commensal bacterium with abundant representation within the entire human skin microbiome, *C. acnes* has been studied very little at the molecular level, and it remains unclear why certain strains of *C. acnes* serve as “skin probiotic” candidates, in contrast to others that have a pathogenic nature and act as opportunistic pathogens in several inflammatory conditions. This can be attributed to the difficulty of culturing *C. acnes*, which is a Gram-positive and anaerobic bacterium that has a lower growth rate than other anaerobic groups (between 5 and 7 days).^4^ By the same token, *C. acnes* is not an easy microorganism to transform or genetically modify, making it more challenging to understand the diverse and complex biological processes that the different strains of *C. acnes* modulate.

On this front, an efficient alternative is the use of an extract-based cell-free system (CFS), which has been described previously for similar challenging organisms.^5,6^ This tool consists of generating an *in vitro* transcription–translation (TX-TL) reaction by mixing crude cell lysate supplemented with an energy buffer, cofactors, and DNA-encoded genetic information (in the form of a plasmid or a linear gene) that will be expressed to create proteins. It is a powerful platform that helps in developing studies in the field of synthetic biology, such as approaches to metabolic engineering that characterize novel enzymes and biosynthetic pathways for biochemical descriptions of novel enzymes in addition to promising models of biosynthetic pathways.^7^

All this can be achieved because of the main advantage of this system, namely, the absence of a cell wall, which allows rapid and direct access to control and manipulate the reaction by direct addition of DNA, thus avoiding arduous cloning and transformation phases. In this case, incubation for only a few hours is needed to test genetic constructs and to have protein expression without purification steps.

To date, the most well studied types of cell-free systems are focused on the platforms of *Escherichia coli*,^8^ wheat germ,^9^ yeast,^10^ and mammalian cells,^11^ all of which have been extensively described and optimized with new methodologies and preparations that have been thoroughly established and validated over time. The most highly developed tool to date is the prokaryotic cell-free system based on *E. coli*,^12,13^ for which two commercial kits are available with promising results for natural product biosynthesis: (a) TX-TL based on a crude cell lysate^14^ and (b) the Protein Synthesis Using Recombinant Elements (PURE) system created from purified proteins (enzymes, ribosomes, and translation factors), which some authors have described as the next-generation cell-free protein system.^15^

However, this *E. coli* platform has some limitations for studying biosynthetic gene clusters from *C. acnes*, as there are several dissimilarities genetically and metabolically: the codon usage, the G + C content, the regulatory genes that modulate transcription, the post-transcriptional modifications, and generally the whole-protein expression and metabolism requirements are different. For this reason, and because of all the advantages that this tool offers, new cell-free systems have emerged in nonmodel organisms such as *Bacillus*,^16^*Streptomyces*,^17^*Vibrio*,^18^ and *Pseudomonas*.^19^

In the present study, we report for the first time the development of a robust *C. acnes* CFS platform that is easy to use in order to accelerate research in the genetic engineering and biotechnology of this microorganism, which has gathered considerable interest in recent years. To achieve this goal, the method of obtaining an active *C. acnes* cell lysis had to be evaluated by testing different strategies to disrupt the bacteria, after which optimization of the energy buffer was performed using the basic *E. coli* CFS with some modifications in order to adjust the final conditions in the reaction, allowing a recombinant firefly luciferase protein yield of up to 85 μg/mL. Furthermore, we include a detailed description of a simple-to-follow protocol for labor-intensive and aerotolerant microorganisms based on three components: lysate, energy buffer, and DNA.

On the other hand, advancements in CFS strategies will allow the design and engineering of new biosynthetic pathways to optimize the production of not only biological materials for tissue engineering but also biofuels and chemicals, thus opening a new door to innovate in sustainable biomanufacturing.^20^ In this *C. acnes* CFS, new approaches such as biofilm engineering properties for future biomaterials would be useful as a cutting-edge strategy for skin tissue remodeling.

It is estimated that this *C. acnes* CFS platform will have a substantial impact in speeding up the design, development, and testing of biological processes in *C. acnes*. Moreover, in terms of applicability, it will foment broad interest in the dermo-cosmetic community to encourage further research on natural products as alternative skin treatments.

## Results and Discussion

Developing a CFS platform from a new microorganism requires the optimization of several steps, including the culture settings, the cell lysis preparation, the composition of the energy buffer, and the final CFS reaction conditions, all of which play important roles in creating a successful reaction. In this research, a cell-free system using methylation-restriction-deficient *C. acnes* KPA171202 was developed for the first time.

### *C. acnes* Lysis Optimization and Evaluation for *In Vitro* TX-TL

To develop a *C. acnes* CFS toolkit for the synthesis of genes involved in skin altered pathways, it was fundamental to optimize how to perform a functional and nondegraded bacterial cell lysis along with a standard protocol to ensure minimal batch variations and high reproducibility.

On the basis of the extensive lysis optimization that had already been established for CFS and for different microorganisms, including mechanical, pressure, acoustic, chemical, and temperature methodologies,^21^ different devices were selected to perform the bacterial lysis, such as a French press, sonicator, or bead homogenizer (MP Biomedicals).^22,23^ However, none of them were successful, as the membrane of the cell envelope of *C. acnes* is not disrupted easily and long lysis times are required because of degradation of the cell lysis components. Therefore, the use of the Freezer Mill instrument was an alternative selected for this purpose. During this lysis process, samples are preserved and frozen with liquid nitrogen, which allows partial removal of the presence of oxygen and thus maintains the anaerobic conditions that this bacterium requires.

**Figure 1:**
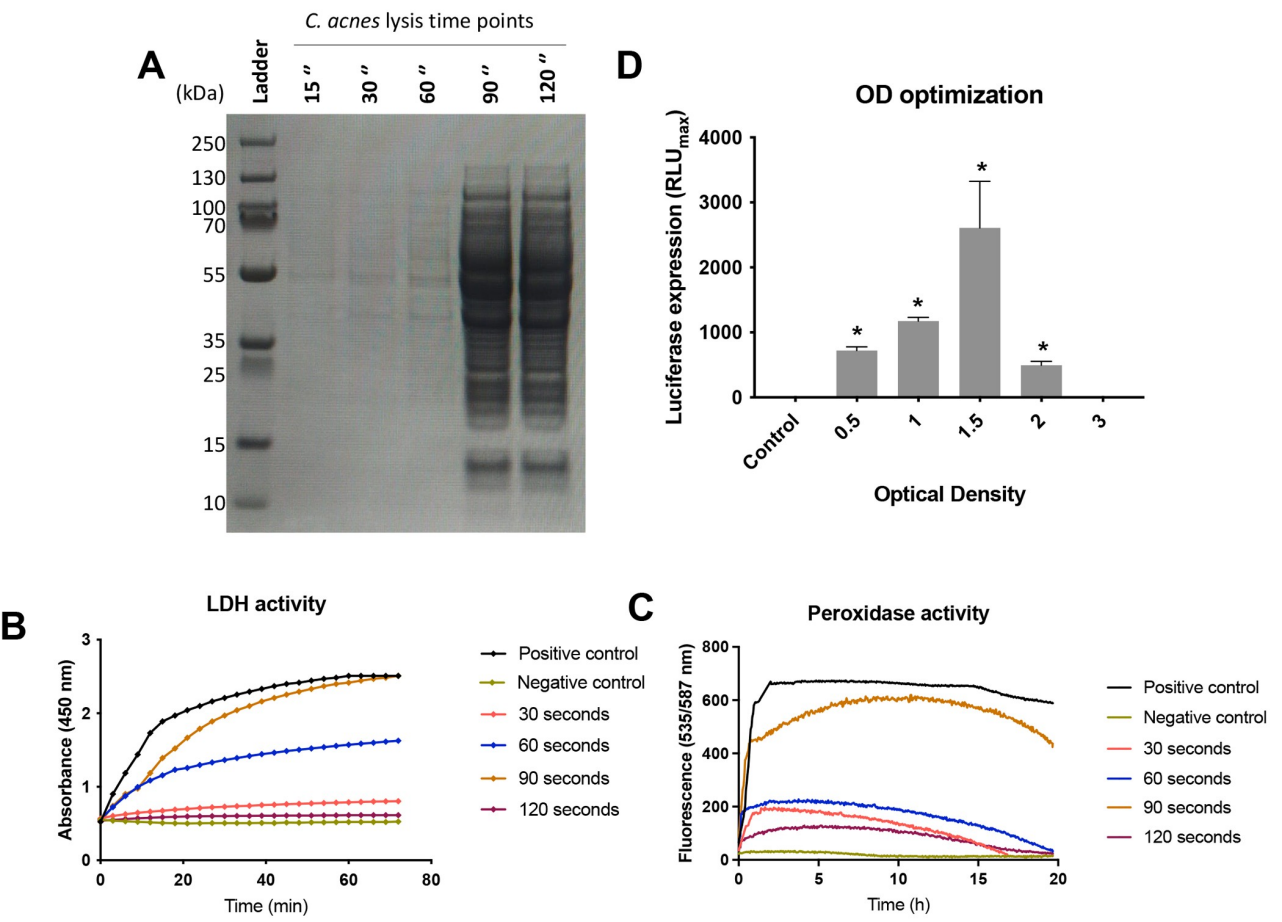
*C. acnes* cell lysis optimization. (A) 10% SDS PAGE at different time points (in s) of lysis of a *C. acnes* pellet using the Freezer Mill at 5 cps. (B) Lactate dehydrogenase (LDH) enzymatic activity measurement during 1 h for different *C. acnes* lysates. (C) Peroxidase enzymatic activity during 20 h for different *C. acnes* lysates. (D) *C. acnes* was cultured in BHI medium to different ODs, and the lysates were tested in the CFS reaction at 30 °C for 24 h for luciferase expression.

To establish the best lysis conditions, the *C. acnes* culture was grown at an optical density at 600 nm (OD_600nm_) of 1, and then the bacterial pellet was harvested under aerobic and sterile conditions by centrifugation. After a three-step wash with sterile phosphate-buffered saline (PBS), the pellet was disrupted after different lysis times (15, 30, 60, 90, and 120 s) but maintaining an energy of 5 cycles per second (cps) in a single cycle. Suspensions were applied to SDS-PAGE gel to distribute the protein content according to the size. As shown by the SDS-PAGE analysis of suspensions presented in Figure 1A, cell lysis for >60 s is required to break down cells, including larger proteins and protein complexes. Therefore, this approach allows extraction of large complexed RNAs like ribosomes, which are essential for the machinery of protein synthesis.^22^

Additionally, on the basis of the experiments by Gannesen et al.,^24^ lysates were also tested for protein functionality as measured by the lactate dehydrogenase (LDH) and peroxidase activities, since both are intracellular enzymes present in *C. acnes*. These results (Figure 1B,C) showed that 90 s is the optimal cell lysis time, showing a higher enzymatic activity for both enzymes and supporting the SDS-PAGE results.

To confirm these results, different cell lysis times were tested in the *C. acnes* CFS reaction using the linear luciferase gene as the reporter (Figure S4).

### *C. acnes* Growth Phase, pH, and Temperature as Key Parameters in the *C. acnes* CFS Reaction

Most studies optimize the cell growth and cell-free protein synthesis conditions as key factors for a successful *in vitro* reaction.^25−28^ In the same way, other parameters including the growth phase, optical density, pH, and temperature play a critical role during the optimization of the CFS reaction.^29,30^ On the basis of previous experiences working with *C. acnes*, it is paramount to highlight the three aforementioned parameters as the inflection points that have a functional reaction.

To carry out the optimization of these parameters, the cell lysis was fixed at 90 s at 5 cps using the Freezer Mill homogenizer. Incubations were performed for 24 h because in a time course of 0, 2, 3, 6, 24, and 48 h, the best time of incubation besides 3 and 6 h was 24 h (Figure S5). The incorporated gene reporter was a linear PCR product containing a T7 promoter and the firefly luciferase gene (Fluc) codon optimized for *C. acnes*, because when the two DNA structures (plasmid and linear gene) were tested in parallel, the linear gene showed a higher trend for luciferase expression (Figure S6). This protein can be measured by a highly sensitive bioluminescent reaction, reducing the background in controls compared with fluorescence reporters.^31^

For the growth phase of the *C. acnes* culture used for the CFS, different OD_600nm_ (0.5, 1, 1.5, 2, and 3) were tested following the previous standardized protocol for the Freezer Mill. Most of the current *E. coli* CFS protocols establish the harvest point by measuring the OD and stopping the bacterial growth in the mid log phase, when the translation machinery is more active and the implicated factors are more abundant.^32^ However, in 2015 Kwon et al. reported that the ideal OD_600nm_ for *in vitro* TX-TL varies depending on the strain,^25^ while Dopp et al. also observed that OD values differ significantly among different types of instruments used as well as among laboratories,^33^ which is vital to ensure the reproducibility of the system. Therefore, in the current study different ODs were adjusted for *C. acnes* measured in 1.5 mm cuvettes using a common spectrophotometer. The results (Figure 1D) showed that a value of 1.5, corresponding to a late-middle exponential phase of growth (Figure S2), had a higher yield for luciferase expression. In contrast, optical densities greater than 2 were associated with the stationary or dead phase, where most of the cellular components were inactive. This is a time-limiting factor for CFS, as *C. acnes* grows very slowly and an OD of 1.5 provides low amounts of pellet. Therefore, to have a proper cell lysis batch, a working bacterial culture volume of 1 L was used.

Moreover, it was observed that *C. acnes* cultures grown without agitation or with optical densities greater than 2 tend to form biofilms and are represented as white aggregates accumulated on the bottom of the flask. In these cases, the bacterial pellet was never functional for the CFS reaction at any point. This can be explained by the reduced presence of bacteria, the altered *C. acnes* metabolism, and the interference of the polysaccharides forming the biofilm.

**Figure 2:**
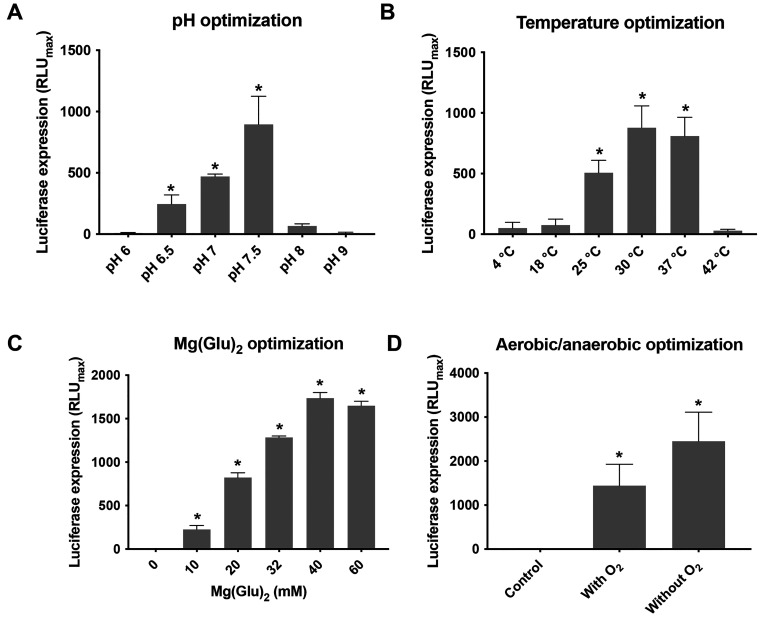
Optimization of key parameters in the *C. acnes* CFS reaction. Different conditions were set up to improve the *C. acnes* in vitro protein production in the CFS reaction. (A) Different reactions were run at different pH values (6, 7.5, 8, and 9) for 24 h at 30 °C, and the luciferase expression was quantified. (B) Different *C. acnes* CFS reactions were carried out at different temperatures (4, 18, 25, 37, and 42 °C) for 24 h, and then the luminescence was measured. (C) Optimization of magnesium requirements in the *C. acnes* CFS reaction to get the best yield of luciferase expression. A range between 20 and 60 mM was tested. (D) The *C. acnes* CFS reaction was run in the presence (tubes closed, trapping the oxygen inside) or absence (tubes opened inside an anaerobic hood) of oxygen for 24 h at 30 °C. The results are presented as mean ± standard deviation (SD) of triplicate measurements (*p* < 0.05) vs a negative control (CFS reaction without DNA).

The importance of the pH in bacterial metabolic pathways has been well-studied for different microorganisms. In human skin, *C. acnes* lives in a low to medium pH environment (between 5 and 7.4), due mainly to the secretion of short-chain fatty acids, among others. However, the intracellular machinery of this bacterium works with higher pH than the physiological pH (between 7 and 9).^34^ These data were relevant for the CFS optimization, and on the basis of this information, different pH-adjusted reactions with NaOH were performed in parallel for 24 h at 30 °C. The CFS reaction without pH modification had a value of 6.5, whereas the adjusted reactions had final pH values of 7, 7.5, 8, and 9. The results in Figure 2A demonstrate that a pH lower than 7 or higher than 8 inhibits protein production. Therefore, it was determined that the optimal pH for *in vitro* TX-TL reactions in *C. acnes* is 7.5.

With regard to temperature, previous work demonstrated that 30 °C provides an optimal balance of nuclease and protease activity along with metabolic and protein synthesis activity, ensuring maximum protein expression.^17,35^ Therefore, different temperatures were tested for the *C. acnes* CFS including 4, 18, 25, 30, and 37 °C. After 24 h of reaction, the results for luciferase production showed that 30 °C gave the maximum expression, followed by 37 °C, whereas 18 and 25 °C gave values comparable to that for the negative control (CFS reaction without DNA) (Figure 2B).

### High Concentrations of Mg(Glu)_2_ and the Absence O_2_ Enhance Protein Expression in the *C. acnes* CFS

It is well-described in the literature that magnesium is an essential component for biochemical activity of eukaryotic cells, being necessary as a cofactor for ribozymes for processing and cleavage of mRNA.^36^ In the same way, magnesium is required to start bacterial translation, as it neutralizes hydrogen bonds in the structure of the rRNA, stabilizing the ribosomal scaffold and allowing protein synthesis.^37^ Additionally, magnesium is required for the CFS machinery, and the accumulation of inorganic phosphate sequesters it, blocking the protein synthesis.^38^ Therefore, a critical factor in most of the studies involving CFS platforms is the optimization of magnesium in the final reaction to increase the productivity.^25,35,39,40^ On the basis of this information, the *C. acnes* CFS reaction was set up over a range of different concentrations of Mg(Glu)_2_ between 10 and 60 mM. As Figure 2C shows, concentrations higher than 10 mM increase the luciferase expression remarkably, and the production was optimal at 40 mM Mg(Glu)_2_. Similar results were also shown in the only publication describing a CFS for anaerobic bacteria,^35^ in contrast to the rest of the TX-TL described for other microorganisms, including *E. coli*, where the optimal magnesium concentrations are between 8 and 12 mM.^25,41^ This could be explained as a different metabolic mechanism to recycle the free Mg^2+^ in accordance with the bacterial type.^42^ In this regard, it is likely that the bacterial machinery uses the associated glutamate as a fermentation pathway, resulting in better use of the energy resources.^43^ However, further studies are needed to more comprehensively understand the high requirement of magnesium for protein synthesis in this *C. acnes* CFS.

With regard to the anaerobic conditions for *in vitro* protein expression, a new way to improve the *E. coli* synthesis capacity in a CFS platform was recently described in which the amount of dissolved oxygen present in the reaction is reduced. This environment would force the anaerobic metabolism of glucose into pyruvate, which apparently speeds up the machinery for protein synthesis, thus increasing the yield.^44^ In this context, the *C. acnes* CFS was tested in both aerobic and anaerobic environments for 24 h at 30 °C with the luciferase gene included. As Figure 2D shows, the depletion of oxygen tends to increase the luciferase expression. These results support the previous data reported by Tamiev et al.^44^ and the importance of anaerobic reactions, opening a new path to improve and optimize other CFSs that have already been established. The obtained results displayed that *C. acnes* TX-TL reactions have a maximum protein yield of 85 μg/mL, a figure that is considered low compared with other atypical *E. coli* CFSs that have already been reported to have averages between 190 and 250 μg/mL.^17,35,45,46^ These data suggest that for a first approach to a CFS using a fastidious microorganism as *C. acnes*, the optimized platform would be able to produce a medium yield. However, further studies are necessary that focus on finding new, strong promoters or optimize semicontinuous models that allow the system to increase the protein synthesis production.

### Screening of *C. acnes* Promoters by Detection of *In Vitro* Transcription in the CFS Reaction

The rapid development of cell-free systems for nonmodel organisms has led to the creation of new methodologies for rapid screening of results in parallel. One of those techniques used by several authors is quantification of mRNA expressed in the *in vitro* reaction by means of reverse transcription–quantitative polymerase chain reaction (RT-qPCR), which is directly related to the activity of transcription in the TX-TL system (Chen and Lu,^47^ Choi et al.,^48^ and Moore et al.^16^).^49^

**Figure 3:**
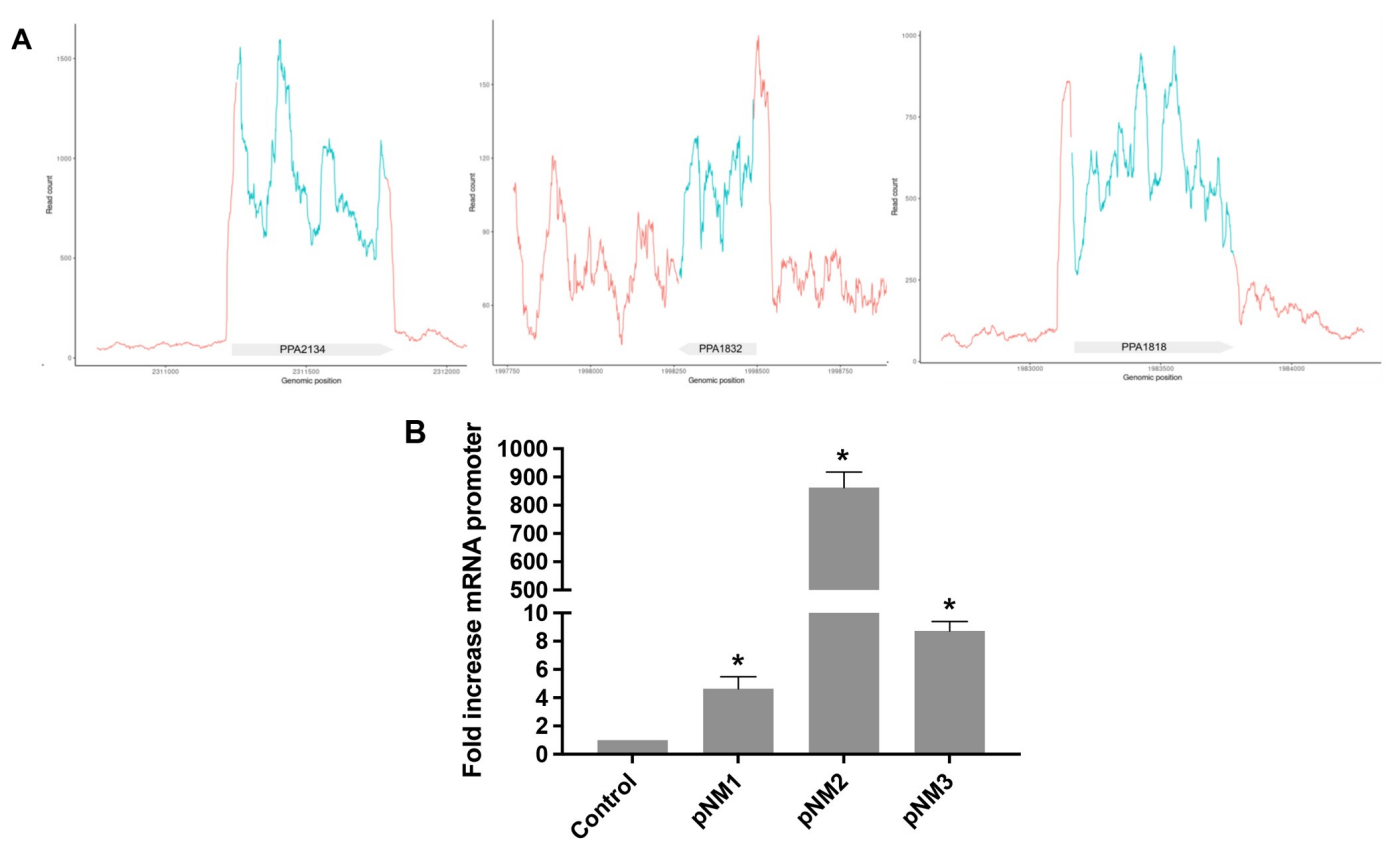
CFS transcriptomic analysis and screening of *C. acnes* promoters in the CFS reaction by RT-qPCR. (A) Representative RNa-seq results for highly expressed cytosolic proteins that are the first in the operon and smaller than 650 nucleotides. Reads for all three samples are aligned to the reference genome. The panels show from top to bottom the coverage, reads, and genomic annotations from three samples. Three panels are sorted according to strength of expression: PPA2134 (DNA starvation protein), PPA1832 (translation initiation factor IF-1) and PPA1818 (superoxide dismutase). (B) Plasmids containing PPA1832 (pNM2), PPA2134 (pNM3) and PPA1818 (pNM1), were assayed in the *C. acnes* CFS reaction by incubation for 3 h at 30 °C. Then RNA was extracted, treated with DNase I, and retrotranscribed for evaluation of the promoter expression level by qPCR. The results are presented as mean ± SD of triplicate measurements (*p* < 0.05) vs a negative control (CFS reaction without DNA).

In this study, several putative endogenous promoters for *C. acnes* were selected to be tested: PPA1818 (carried on plasmid pNM1), PPA1832 (pNM2), and PPA2134 (pNM3). As Figure 3A shows, RNA sequencing (RNA-seq) read counts in each genomic position show a high number of mapped reads (between 150 and 1000) in these three transcripts. Additionally, there is a large divergence between the absolute numbers of counts in the annotated region and the surrounding region. This abrupt change is clearer with the gene annotation position of PPA2134, while gene annotations are shifted downstream of the sequence for PPA1818 and PPA1832. The background signal is high in the region where PPA1832 is located, while PPA2134 shows high expression without surrounding noise.

In the three different CFS reactions (pNM1, pNM2, and pNM3), after incubation for 3 h at 30 °C, the RNA was isolated, and the mRNA expression level of each promoter was quantified. A critical step in this methodology involves the use of DNase I treatment to ensure the degradation of the original DNA added into the reaction in order to avoid false positive results. As Figure 3B shows, all three promoters tested were functional in the *C. acnes* CFS but not with the same intensity, as PPA1832 was highly expressed compared with the others, meaning that it could be used as a strong promoter for future engineering developments and helpful in finding new biosynthesis pathways associated with this strain.

In parallel, using the linear construct T7-Fluc, the correlation between mRNA and protein expression measured by luminescence was measured to validate the system. As Figure S8 shows, the greater mRNA expression resulted in a greater protein level. Therefore, this platform is a potential toolkit to validate functionality of putative genes previously selected by transcriptomic analysis.

### *C. acnes* CFS as a Rapid Platform to Screen Promoters (Biosensors)

**Figure 4:**
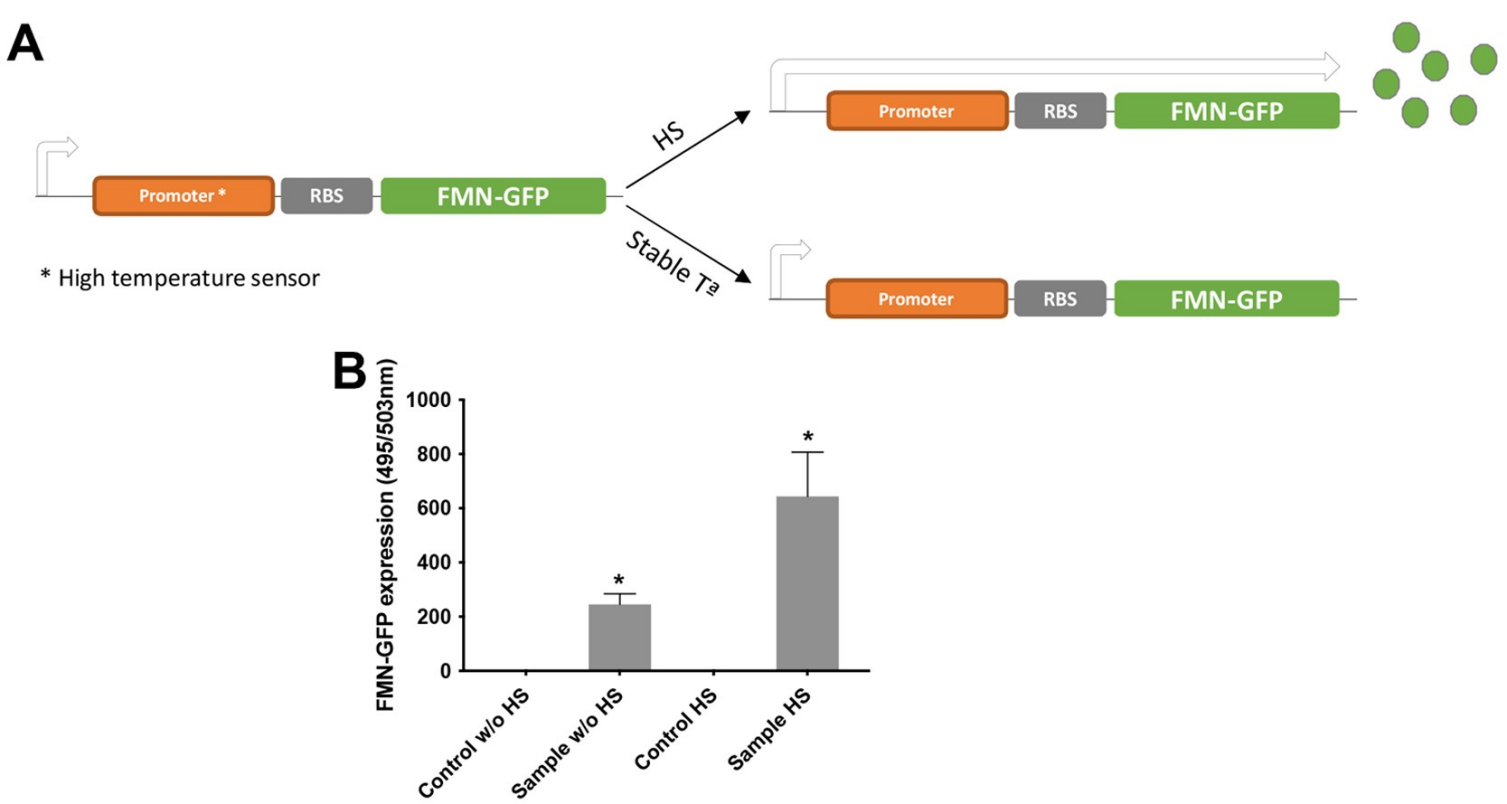
*C. acnes* CFS as a platform to test a *C. acnes* promoter acting as a temperature biosensor. (A) Scheme of how the plasmid pNA14 containing the thermosensor promoter codified by the PPA1344 gene works. (B) After 1 h at 30 °C, some CFS reactions were incubated for 2 min at 42 °C to provide a heat shock (HS), and in parallel, negative control (without DNA) samples were maintained at 30 °C. After incubation for 24 h, FMN-GFP protein expression was measured at 495/503 nm. Results are presented as mean ± SD of triplicate measurements (*p* < 0.05) vs a negative control (CFS reaction without DNA).

The CFS technique is a fast protein expression system that allows testing of different bacterial gene constructs at the same time; thus, these characteristics make it suitable for testing sensor candidates acting as cell-free biosensors of high impact in biomedical and cosmetic applications.^50^ In this context, the *C. acnes* CFS was used to assay the pNA14 plasmid containing the flavin mononucleotide–green fluorescent protein (FMN-GFP) gene reporter controlled by a modulable promoter with high activity under high-temperature conditions (Figure 4A).

FMN-GFP was selected as a reporter in the *C. acnes* CFS, as opposed to the commonly used GFP, because FMN-GFP is active even under anaerobic conditions. As was previously reported, low levels of oxygen improve the CFS reaction, and therefore, this version of fluorescence was a better candidate to be tested. To activate this promoter in the CFS reaction, a heat shock (HS) of 42 °C for 2 min was applied, while the rest of the incubation period was held at a constant temperature of 30 °C. In parallel, the normal T7 promoter–luciferase linear gene was evaluated as a control for the whole system (Figure S9). After 24 h, higher FMN-GFP protein expression was observed in those samples containing the gene construct compared with both negative controls (without DNA). However, the sample submitted to HS had a higher protein expression than that without the HS treatment (Figure 4B). In the case of the T7-luciferase construct, only the reaction without the HS was shown to be functional, which can be explained by loss of the activity of the T7 promoter/T7 RNA polymerase complex at high temperatures.^51^ These results provide evidence that the CFS system is indeed able to validate the differential gene expression of a *C. acnes* promoter in a relevant skin signal (temperature). The same platform would also be useful in validating and characterizing genes with differential expression under other relevant skin conditions and metabolites such as the presence of hormones, serum production, or inflammation (oxidative stress). Once these sequences are validated, they become great candidates to develop genetic circuits within *C. acnes* to develop skin biosensors.

## Conclusions

In recent times there has been significant interest in the use of cell-free system platforms from model and nonmodel organisms as means to study biosynthetic pathways or to produce recombinant proteins using a quick and uncomplicated technique.^17,19,35,45,46^ In this context, the present study provides new information with regard to the development of a CFS toolkit for *Cutibacterium acnes*, which is one of the most abundant microorganisms in human skin microbiota and is involved in several skin alterations. To do so, the cell lysis protocol was optimized, as were key parameters such as pH, temperature, and optical density. Moreover, *C. acnes* CFS activity can be improved with high doses of Mg(Glu)_2_, as was previously described for an anaerobic *Clostridium* strain, and also maintaining the absence of oxygen (anaerobic conditions) during the *in vitro* reaction. The final TX-TL toolkit was able to produce around 85 μg/mL luciferase within a 24 h batch reaction, making it the first *C. acnes* CFS described to date. The data also demonstrated that the *C. acnes* CFS developed is an appealing alternative for screening of putative genes that saves time and effort while using routine techniques, especially when working with time-consuming microorganisms that must be cultivated. Another useful application is the use of the *C. acnes* CFS as a biosensor, as its functionality was proven with a temperature-modulable promoter. These results demonstrate the early-stage potential of the *C. acnes* TX-TL to study the genetic pathways and metabolites that *C. acnes* regulates on human skin and the applicability of the biosensor to treat or prevent certain skin diseases such as acne vulgaris.

## Materials and Methods

### Bacterial Strain

In this study, the strain used was a mutant of the common *Cutibacterium acnes* DSM 16379/KPA171202 (DSMZ-German Collection of Microorganisms and Cell Cultures GmbH). The wild-type strain was genetically modified to create a strain lacking active restriction modification systems to prevent DNA degradation and improve protein production. Strains were commonly grown in brain heart infusion (BHI) broth (Millipore) to exponential phase. The mutant strain was supplemented with erythromycin 10 μg/mL).

### Production of a Restriction-Modification-Deficient KPA171202 Mutant Strain

We followed a strategy previously described by Sörensen et al.^52^ to create a methylation-deficient KPA171202 strain. In summary, we amplified 500 bp upstream (primers 468/469) and downstream (primers 470/471) of the locus to be replaced, ligated them, and cloned them into the pGEM-T-easy vector. In a second step, the erythromycin cassette was cloned between the homology arms using Acc65I restriction sites and positive clones selected to be transformed into dam-*E. coli* strain GM2199. *C. acnes*-competent cells were prepared as previously described,^52^ and 8 μg of the plasmid DNA containing the homology arms and erythromycin cassette was transformed and selected on Brucella agar plates containing 10 μg/mL erythromycin. After 7 days of anaerobic growth, colonies were screened for deletion of the R-M locus and for genomic insertion of the erythromycin cassette using junction PCR primer pairs (499/338) and (500/308). Correct knockout was additionally verified by whole-genome sequencing.

### Identification of Putative Promoters by Transcriptomic Analysis in *C. acnes* CFS

Candidate promoters were selected after analysis of the RNA-seq data obtained from a 24 h *C. acnes* CFS reaction. Fastq files were aligned against the *Propionibacterium acnes* AE017283.1 reference genome with BWA-MEM. Samtools were used to convert the sam file into a bam file and sort the alignment. A custom R script was used to calculate transcripts per million (TPM). TPM was calculated by multiplying the number of reads mapped to a particular transcript by 1 million and dividing by the length of the transcript. Among the top expressed genes, other parameters were used to select potential strong promoters. We filtered out all noncoding genes, hypothetical genes, genes longer than 500 bp, genes that were not first in the operon, and noncytoplasmatic proteins.

The candidate promoter expression profile was obtained from the pileup. RNA-seq reads were aligned against the *P. acnes* AE017283.1 reference genome with BWA-MEM. Samtools were used to convert the sam file into a bam file, sort the alignment, and finally get the pileup file with mpileup. The pileup file was used to directly plot the count of reads per position. In addition, The GeneBank annotation file of the reference genome was used to retrieve the candidate gene and 500 nucleotides on each side to zoom in the regions of interest.

### Reporter Construct Generation

To optimize the CFS reaction, two main genes were used as reporters driven by a T7 promoter: luciferase and flavin mononucleotide-based green fluorescent protein (FMN-GFP). Both of them were codon-optimized for *C. acnes* and synthesized by Twist Biosciences.

The final construct tested in the reaction involved linear genes (PCR-purified) or plasmids (Golden Gate assembly from NEB). For PCR, KAPA Hifi Polymerase (Roche) was used, following the cycling protocol recommended by the manufacturer. For cloning, the pUC19-derived vector was used, and sequences were confirmed by Sanger sequencing. To create a promoter library, we chose three highly expressed, short in sequence, first in operon cytosolic proteins identified by RNaseq, including PPA1818 (pNM1), PPA1832 (pNM2), and PPA2134 (pNM3). All three endogenous genes with a C-terminal his-taq were cloned into the pUC19 backbone by Golden Gate assembly using BspQI restriction sites. Afterward those genes were added into the CFS mix, and transcription was measured by qPCR using primers pNM1_qPCR_fwd, pNM2_qPCR_fwd, pNM3_qPCR_fwd, and pNM_qPCR_rev.

Moreover, the endogenous gene PPA1344, which was identified as a putative temperature sensor after exposure of *C. acnes* to a heat shock for 15 min at 42 °C and analysis of the information by RNaseq, was tested as a biosensor in the CFS reaction. Therefore, pNA14 was built using a previously described modular assembly strategy.^53^ For that, 200 base pairs upstream of PPA1344 containing the regulatory elements of the promoter were amplified with GN_225 and GN_226 from the *C. acnes* genome and cloned into a modified version of pJET1.2/blunt (Thermo Fisher) storage plasmid. Then the PPA1244 promoter was cloned upstream of a strong ribosome binding site followed by an anaerobic FbFp reporter.^54^ Both parts were amplified from the storage vectors to include appropriate overlaps using the primer pairs PR-UNA/PR-D17 and PR-U17/PR-DNA, respectively, and introduced in the pTU-A backbone using Gibson assembly to create the final pNA14. All of the mentioned plasmids were transformed in NZYa *E. coli* cells (NZYTech) that were chemically competent for cloning purposes.

The different constructs generated for this strain are listed in Tables S2 and S3.

### *C. acnes* Growth Conditions and Harvesting

*C. acnes* was grown in sterile 75 cm^2^ flaks (Sigma-Aldrich, SIAL0641) with a working volume of 100 mL of BHI broth. To keep anaerobic conditions, the flask was placed in a 2.5 L anaerobic jar with the Oxoid AnaeroGen system. Incubation was performed at 37 °C with shaking at 110 rpm. The initial OD_600nm_ was set up at 0.05, and after different times of incubation (24, 42, 55, 65, and 96 h), different optical densities were obtained (0.5, 1, 1.5, 2, and 3, respectively). The rest of the stages were performed in the presence of oxygen. The cultures were centrifuged at 2500*g* for 10 min, and the supernatant was discarded by decantation. The pellet was washed three times with 50 mL of sterile HBSS solution (Gibco). Residual buffer was removed with a pipet after 1 min of centrifugation at 10000*g*. The bacterial mass pellet was weighed, directly transferred into liquid N_2_, and stored at −80 °C.

### Cell Lysis Preparation

*C. acnes* pellets were thawed on ice and resuspended in sterile S30 buffer (10 mM Tris-acetate, pH 8.2, 14 mM magnesium acetate, 10 mM potassium acetate, and 4 mM DTT added new every time). The proportion used was 0.33 v/w. After vortexing, the homogeneous solution was pipetted into liquid N_2_, forming small beads. Next, 0.5 g of beads was lysed with the Freezer Mill (SPEX SamplePrep) under different setup conditions: 1 cycle at 5 cps for 15, 30, 60, and 90 s. Then the powder lysate was transferred into 50 mL tubes, thawed in a water bath at 30 °C for 1 min with continuous mixing, and centrifuged at 12000*g* and 4 °C for 10 min to clarify the lysate. Then 50 μL of the soluble fraction was seeded on a Brucella agar plate to test for bacterial contamination (Figure S1). The rest was transferred into new 1.5 mL tubes, and the centrifugation step was repeated. The clear supernatant was quantified for total protein amount with the BCA assay (Thermo Fisher), and only values between 14 and 19 mg/mL were active for the CFS reaction. Then aliquots were made, frozen in liquid N_2_, and stored immediately at −80 °C until use.

Total protein distribution was visualized by sodium dodecyl sulfate polyacrylamide gel electrophoresis (SDS-PAGE). To do that, 10 μL of the CFS reaction was mixed with loading buffer and incubated at 98 °C for 5 min. Then samples were run for 80 min at 100 V. Proteins were stained with Coomassie Brilliant Solution (BioRad) for 30 min and destained overnight with a solution of water, methanol, and acetic acid (50/40/20 v/v/v).

### Enzymatic Activity to Evaluate Bacterial Lysis

To prove the integrity and biofunctionality of the protein content of the different *C. acnes* lysates, they were tested for two enzymatic activities: lactate dehydrogenase and peroxidase (Sigma-Aldrich) following the manufacturer’s instructions. In both assays, 40 μg samples of total protein of *C. acnes* lysates were used in a final volume of 50 μL in 96-well plates incubated at 37 °C.

### CFS Reaction Conditions

Following the protocol described by Krüger et al.^35^ and supplementing it with specific components used in the *Streptomyces* CFS protocol reported by Moore et al.,^17^ the final components in our the CFS reaction mixture included 50% cell lysate, 40% energy buffer, and 10% DNA. For the energy buffer, a 20× working solution was prepared with the following concentrations in the final reaction: 1.2 mM nucleotides (ATP, GTP, UTP, and CTP), 0.069 mM folinic acid, 0.17 mg/mL *E. coli* tRNA, 1 mM amino acids, 0.33 mM NAD, 0.27 nM CoA, 1.5 mM spermidine, 1 mM putrescine, 4 mM sodium oxalate, 32 mM magnesium glutamate, 150 mM potassium glutamate, 57 mM HEPES (pH 8), 33 mM PEP, 30 mM 3-PGA, 1% w/v PEG6K, 16 μg/mL T7 RNA polymerase, 1.5 μg GamS nuclease inhibitor (NEBExpress P0774S), and 1 μL of 50× phosphatase inhibitor cocktail (Promega G6521). The energy solution was completed with 8 mg/mL lysate and 40 nM DNA template (Table S1). The final reaction mixture was adjusted to the specified pH (6, 7.5, 8, or 9) with a sterile 1 M NaOH solution. The working volume for the final mixture was 50 μL in 1.5 mL tubes. Reactions were carried out in an Eppendorf ThermoMixer with shaking at 800 rpm at different temperatures (4, 16, 25, 30, 37, and 42 °C). The best conditions selected for CFS reactions were run in parallel inside of an anaerobic chamber. In all cases, at least three independent batches of lysates were assayed on separate days.

### RT-qPCR Assay

To validate the *in vitro* transcription for the *C. acnes* CFS, after 3 h of reaction, total RNA was isolated using a miRNeasy Kit (Qiagen) following the manufacturer’s instructions, including the DNase treatment to avoid false positives. The RNA concentration and quality were tested using the NanoDropTM (Thermo Fisher). A 500 ng sample of RNA was reverse-transcribed into cDNA using the Revertaid First Strand cDNA Synthesis Kit (Thermo Fisher). After that, qPCR was carried out in a QuantStudio Thermocycler (Applied Biosystems). The primers used are listed in Table S1. The relative gene expression was normalized with the housekeeping RecA gene, and the formula 2^–ΔΔCt^ was applied.

### Luminescence and Fluorescence Quantification

For luminescence, firefly luciferase expression was determined using the Luciferase Assay System (Promega), an Infinite 200 PRO NanoQuant microplate reader (Tecan), and 384-well black flat-bottom plates (Corning). The assay was performed by mixing 5 μL of the CFS reaction mixture and 25 μL of luciferase substrate just before the plate was run. Luminescence data, as maximum amounts of relative light units (RLUs), were collected every 30 s over a 10 min time period. Purified luciferase was obtained from Sigma-Aldrich to estimate the protein concentration through a standard curve extrapolation (Figure S3).

To measure FMN-GFP protein fluorescence, 12 μL of the direct CFS reaction mixture was placed in 384-well plates and measured at 495/503 nm.

### *C. acnes* Cell-Free Biosensor Assay

The *E. coli* BL21 containing the plasmid pNA14 was cultured in 5 mL of LB medium at 37 °C. Then the plasmid content was isolated using the Qiagen Miniprep Kit and used for the CFS reaction.

To induce the temperature-modulable promoter, after 1 h at 30 °C the reaction mixtures were incubated for 2 min at 42 °C (heat shock). Negative controls (not including the heat shock) were also performed at the same time. In parallel and as an independent condition, the T7-Fluc construct was tested following the same protocol. After 24 h, all of the samples were measured by luminescence or fluorescence.

### Statistical Analysis

Plots and statistical studies were conducted using GraphPad Prism 7 (GraphPad Software). For parametric analysis of data from quantification of the synthesized protein, two-way ANOVA followed by the Dunnett test was performed. In all cases, a *p* value of 0.05 (5% significance level) was applied.

## Supporting Information Available

Optimized composition of the *C. acnes* CFS reaction (Table S1), detailed sequences of primers and constructs used in this work (Tables S2 and S3), contamination test of *C. acnes* cell lysate (Figure S1), additional information on bacterial growth and setup of *C. acnes* CFS reaction conditions (Figures S3–S6, S8, and S9), and additional information for transcriptomic analysis (Figure S7) (PDF)

## Abbreviations

CFS: cell-free system
LDH: lactate dehydrogenase
FMN-GFP: flavin mononucleotide–green fluorescent protein
TX-TL: transcription–translation
cps: cycles per second
OD: optical density
SDS-PAGE: sodium dodecyl sulfate polyacrylamide gel electrophoresis
RT-qPCR: reverse transcription–quantitative PCR

## References

[ref1] Byrd A. L.; Belkaid Y.; Segre J. A. The Human Skin Microbiome. Nat. Rev. Microbiol. 2018, 16 (3), 143–155. 10.1038/nrmicro.2017.157. 29332945

[ref2] Oh J.; Byrd A. L.; Park M.; Segre J. A. Temporal Stability of the Human Skin Microbiome. Cell 2016, 165 (4), 854–866. 10.1016/j.cell.2016.04.008. 27153496 PMC4860256

[ref3] Paetzold B.; Willis J. R.; Pereira de Lima J.; Knödlseder N.; Brüggemann H.; Quist S. R.; Gabaldón T.; Güell M. Skin Microbiome Modulation Induced by Probiotic Solutions. Microbiome 2019, 7 (1), 95 10.1186/s40168-019-0709-3. 31234928 PMC6591853

[ref4] Hall G. S.; Pratt-Rippin K.; Meisler D. M.; Washington J. A.; Roussel T. J.; Miller D. Growth Curve for *Propionibacterium acnes*. Curr. Eye Res. 1994, 13 (6), 465–466. 10.3109/02713689408999875. 7924410

[ref5] Batista A. C.; Soudier P.; Kushwaha M.; Faulon J.-L. Optimising Protein Synthesis in Cell-Free Systems, a Review. *Eng*. Biol. 2021, 5 (1), 10–19. 10.1049/enb2.12004. PMC9996726 36968650

[ref6] Silverman A. D.; Karim A. S.; Jewett M. C. Cell-Free Gene Expression: An Expanded Repertoire of Applications. Nat. Rev. Genet. 2020, 21 (3), 151–170. 10.1038/s41576-019-0186-3. 31780816

[ref7] Silverman A. D.; Karim A. S.; Jewett M. C. Cell-Free Gene Expression: An Expanded Repertoire of Applications. Nat. Rev. Genet. 2020, 21, 151–170. 10.1038/s41576-019-0186-3. 31780816

[ref8] Jiang N.; Ding X.; Lu Y. Development of a Robust *Escherichia coli*-Based Cell-Free Protein Synthesis Application Platform. Biochem. Eng. J. 2021, 165, 107830 10.1016/j.bej.2020.107830. 33100890 PMC7568173

[ref9] Kanoi B. N.; Nagaoka H.; Morita M.; Tsuboi T.; Takashima E. Leveraging the Wheat Germ Cell-Free Protein Synthesis System to Accelerate Malaria Vaccine Development. Parasitol. Int. 2021, 80, 102224 10.1016/j.parint.2020.102224. 33137499

[ref10] Hodgman C. E.; Jewett M. C. Optimized Extract Preparation Methods and Reaction Conditions for Improved Yeast Cell-Free Protein Synthesis. Biotechnol. Bioeng. 2013, 110, 2643–2654. 10.1002/bit.24942. 23832321

[ref11] Heide C.; Buldum G.; Moya-Ramirez I.; Ces O.; Kontoravdi C.; Polizzi K. M. Design, Development and Optimization of a Functional Mammalian Cell-Free Protein Synthesis Platform. Front. Bioeng. Biotechnol. 2021, 8, 604091 10.3389/fbioe.2020.604091. 33604330 PMC7884609

[ref12] des Soye B. J.; Gerbasi V. R.; Thomas P. M.; Kelleher N. L.; Jewett M. C. A Highly Productive, One-Pot Cell-Free Protein Synthesis Platform Based on Genomically Recoded *Escherichia coli*. Cell Chem. Biol. 2019, 26 (12), 1743–1754. 10.1016/j.chembiol.2019.10.008. 31706984 PMC7008506

[ref13] Garenne D.; Thompson S.; Brisson A.; Khakimzhan A.; Noireaux V. The All-E. ColiTXTL Toolbox 3.0: New Capabilities of a Cell-Free Synthetic Biology Platform. Synth. Biol. 2021, 6 (1), ysab017 10.1093/synbio/ysab017. PMC8546610 34712841

[ref14] Garamella J.; Garenne D.; Noireaux V. TXTL-Based Approach to Synthetic Cells. Methods Enzymol. 2019, 617, 217–239. 10.1016/bs.mie.2018.12.015. 30784403

[ref15] Lavickova B.; Maerkl S. J. A Simple, Robust, and Low-Cost Method to Produce the PURE Cell-Free System. ACS Synth. Biol. 2019, 8 (2), 455–462. 10.1021/acssynbio.8b00427. 30632751

[ref16] Moore S. J.; MacDonald J. T.; Wienecke S.; Ishwarbhai A.; Tsipa A.; Aw R.; Kylilis N.; Bell D. J.; McClymont D. W.; Jensen K.; Polizzi K. M.; Biedendieck R.; Freemont P. S. Rapid Acquisition and Model-Based Analysis of Cell-Free Transcription–Translation Reactions from Nonmodel Bacteria. Proc. Natl. Acad. Sci. U.S.A. 2018, 115 (19), E4340–E4349. 10.1073/pnas.1715806115. 29666238 PMC5948957

[ref17] Moore S. J.; Lai H. E.; Chee S. M.; Toh M.; Coode S.; Chengan K.; Capel P.; Corre C.; de Los Santos E. L. C.; Freemont P. S. A *Streptomyces venezuelae* Cell-Free Toolkit for Synthetic Biology. ACS Synth. Biol. 2021, 10 (2), 402–411. 10.1021/acssynbio.0c00581. 33497199 PMC7901020

[ref18] Zhu B.; Gan R.; Cabezas M. D.; Kojima T.; Nicol R.; Jewett M. C.; Nakano H. Increasing Cell-Free Gene Expression Yields from Linear Templates in *Escherichia coli* and *Vibrio natriegens* Extracts by Using DNA-Binding Proteins. Biotechnol. Bioeng. 2020, 117 (12), 3849–3857. 10.1002/bit.27538. 32816360

[ref19] Wang H.; Li J.; Jewett M. C. Development of a *Pseudomonas putida* Cell-Free Protein Synthesis Platform for Rapid Screening of Gene Regulatory Elements. Synth. Biol. 2018, 3 (1), ysy003 10.1093/synbio/ysy003. PMC7445763 32995512

[ref20] Kelwick R. J. R.; Webb A. J.; Freemont P. S. Biological Materials: The Next Frontier for Cell-Free Synthetic Biology. Front. Bioeng. Biotechnol. 2020, 8, 399 10.3389/fbioe.2020.00399. 32478045 PMC7235315

[ref21] Cole S. D.; Miklos A. E.; Chiao A. C.; Sun Z. Z.; Lux M. W. Methodologies for Preparation of Prokaryotic Extracts for Cell-Free Expression Systems. Synthetic and Systems Biotechnology 2020, 5 (4), 252–267. 10.1016/j.synbio.2020.07.006. 32775710 PMC7398980

[ref22] Shrestha P.; Holland T. M.; Bundy B. C. Streamlined Extract Preparation for *Escherichia coli*-Based Cell-Free Protein Synthesis by Sonication or Bead Vortex Mixing. BioTechniques 2012, 53 (3), 163–174. 10.2144/0000113924. 22963478

[ref23] Sun Z. Z.; Hayes C. A.; Shin J.; Caschera F.; Murray R. M.; Noireaux V. Protocols for Implementing an *Escherichia coli* Based TX-TL Cell-Free Expression System for Synthetic Biology. J. Visualized Exp. 2013, 79 (No), 50762 10.3791/50762. PMC3960857 24084388

[ref24] Gannesen A. V.; Zdorovenko E. L.; Botchkova E. A.; Hardouin J.; Massier S.; Kopitsyn D. S.; Gorbachevskii M. V.; Kadykova A. A.; Shashkov A. S.; Zhurina M. V.; Netrusov A. I.; Knirel Y. A.; Plakunov V. K.; Feuilloley M. G. J. Composition of the Biofilm Matrix of *Cutibacterium acnes* Acneic Strain RT5. Front. Microbiol. 2019, 10, 1284 10.3389/fmicb.2019.01284. 31293526 PMC6598116

[ref25] Kwon Y. C.; Jewett M. C. High-Throughput Preparation Methods of Crude Extract for Robust Cell-Free Protein Synthesis. Sci. Rep. 2015, 5 (1), 1–8. 10.1038/srep08663. PMC4345344 25727242

[ref26] Silverman A. D.; Kelley-Loughnane N.; Lucks J. B.; Jewett M. C. Deconstructing Cell-Free Extract Preparation for in Vitro Activation of Transcriptional Genetic Circuitry. ACS Synth. Biol. 2019, 8 (2), 403–414. 10.1021/acssynbio.8b00430. 30596483 PMC6584022

[ref27] Carlson E. D.; Gan R.; Hodgman C. E.; Jewett M. C. Cell-Free Protein Synthesis: Applications Come of Age. Biotechnol. Adv. 2012, 30 (5), 1185–1194. 10.1016/j.biotechadv.2011.09.016. 22008973 PMC4038126

[ref28] Gregorio N. E.; Levine M. Z.; Oza J. P. A User’s Guide to Cell-Free Protein Synthesis. Methods Protoc. 2019, 2 (1), 24 10.3390/mps2010024. 31164605 PMC6481089

[ref29] Seki E.; Matsuda N.; Yokoyama S.; Kigawa T. Cell-Free Protein Synthesis System from *Escherichia coli* Cells Cultured at Decreased Temperatures Improves Productivity by Decreasing DNA Template Degradation. Anal. Biochem. 2008, 377 (2), 156–161. 10.1016/j.ab.2008.03.001. 18375196

[ref30] Caschera F.; Noireaux V. Synthesis of 2.3 Mg/Ml of Protein with an All *Escherichia coli* Cell-Free Transcription-Translation System. Biochimie 2014, 99 (1), 162–168. 10.1016/j.biochi.2013.11.025. 24326247

[ref31] Troy T.; Jekic-McMullen D.; Sambucetti L.; Rice B. Quantitative Comparison of the Sensitivity of Detection of Fluorescent and Bioluminescent Reporters in Animal Models. Mol. Imaging 2004, 3 (1), 153535002004031 10.1162/15353500200403196. 15142408

[ref32] Bosdriesz E.; Molenaar D.; Teusink B.; Bruggeman F. J. How Fast-Growing Bacteria Robustly Tune Their Ribosome Concentration to Approximate Growth-Rate Maximization. FEBS J. 2015, 282 (10), 2029–2044. 10.1111/febs.13258. 25754869 PMC4672707

[ref33] Myers J. A.; Curtis B. S.; Curtis W. R. Improving Accuracy of Cell and Chromophore Concentration Measurements Using Optical Density. BMC Biophys. 2013, 6 (1), 4 10.1186/2046-1682-6-4. 24499615 PMC3663833

[ref34] Futsaether C. M.; Kjeldstad B.; Johnsson A. Measurement of the Intracellular pH of *Propionibacterium acnes*: Comparison between the Fluorescent Probe BCECF and ^31^P-NMR Spectroscopy. Can. J. Microbiol. 1993, 39 (2), 180–186. 10.1139/m93-025. 8467419

[ref35] Krüger A.; Mueller A. P.; Rybnicky G. A.; Engle N. L.; Yang Z. K.; Tschaplinski T. J.; Simpson S. D.; Köpke M.; Jewett M. C. Development of a Clostridia-Based Cell-Free System for Prototyping Genetic Parts and Metabolic Pathways. Metab. Eng. 2020, 62, 95–105. 10.1016/j.ymben.2020.06.004. 32540392

[ref36] Vernon W. B. The Role of Magnesium in Nucleic-Acid and Protein Metabolism. Magnesium 1988, 7 (5–6), 234–248. 2472534

[ref37] Laursen B. S.; Sørensen H. P.; Mortensen K. K.; Sperling-Petersen H. U. Initiation of Protein Synthesis in Bacteria. Microbiol. Mol. Biol. Rev. 2005, 69 (1), 101–123. 10.1128/MMBR.69.1.101-123.2005. 15755955 PMC1082788

[ref38] Kim T. W.; Kim D. M.; Choi C. Y. Rapid Production of Milligram Quantities of Proteins in a Batch Cell-Free Protein Synthesis System. J. Biotechnol. 2006, 124 (2), 373–380. 10.1016/j.jbiotec.2005.12.030. 16487613

[ref39] Li J.; Wang H.; Jewett M. C. Expanding the Palette of *Streptomyces*-Based Cell-Free Protein Synthesis Systems with Enhanced Yields. Biochem. Eng. J. 2018, 130, 29–33. 10.1016/j.bej.2017.11.013.

[ref40] des Soye B. J.; Davidson S. R.; Weinstock M. T.; Gibson D. G.; Jewett M. C. Establishing a High-Yielding Cell-Free Protein Synthesis Platform Derived from *Vibrio natriegens*. ACS Synth. Biol. 2018, 7 (9), 2245–2255. 10.1021/acssynbio.8b00252. 30107122

[ref41] Jewett M. C.; Swartz J. R. Mimicking the *Escherichia coli* Cytoplasmic Environment Activates Long-Lived and Efficient Cell-Free Protein Synthesis. Biotechnol. Bioeng. 2004, 86 (1), 19–26. 10.1002/bit.20026. 15007837

[ref42] Webb M. The Utilization of Magnesium by Certain Gram-Positive and Gram-Negative Bacteria. J. Gen. Microbiol. 1966, 43, 401–409. 10.1099/00221287-43-3-401. 4960404

[ref43] Buckel W.; Barker H. A. Two Pathways of Glutamate Fermentation by Anaerobic Bacteria. J. Bacteriol. 1974, 117 (3), 1248–1260. 10.1128/jb.117.3.1248-1260.1974. 4813895 PMC246608

[ref44] Tamiev B. D.; Dopp J. L.; Reuel N. F. Anaerobic Conditioning of *E. coli* Cell Lysate for Enhanced in Vitro Protein Synthesis. ACS Synth. Biol. 2021, 10 (4), 716–723. 10.1021/acssynbio.0c00501. 33760595 PMC8168642

[ref45] Kelwick R.; Webb A. J.; MacDonald J. T.; Freemont P. S. Development of a *Bacillus subtilis* Cell-Free Transcription-Translation System for Prototyping Regulatory Elements. Metab. Eng. 2016, 38, 370–381. 10.1016/j.ymben.2016.09.008. 27697563

[ref46] Wiegand D. J.; Lee H. H.; Ostrov N.; Church G. M. Cell-Free Protein Expression Using the Rapidly Growing Bacterium *Vibrio natriegens*. J. Visualized Exp. 2019, (No), 145 10.3791/59495. PMC6512795 30933074

[ref47] Chen X.; Lu Y. In Silico Design of Linear DNA for Robust Cell-Free Gene Expression. Front. Bioeng. Biotechnol. 2021, 9, 670341 10.3389/fbioe.2021.670341. 34095101 PMC8169995

[ref48] Choi Y.-N.; Shin Y. R.; Park J. M.; Lee J. W. Cell-Free Transcription-Coupled CRISPR/Cas12a Assay for Prototyping Cyanobacterial Promoters. ACS Synth. Biol. 2021, 10 (6), 1300–1307. 10.1021/acssynbio.1c00148. 34015913

[ref49] Failmezger J.; Rauter M.; Nitschel R.; Kraml M.; Siemann-Herzberg M. Cell-Free Protein Synthesis from Non-Growing, Stressed *Escherichia coli*. Sci. Rep. 2017, 7 (1), 1–10. 10.1038/s41598-017-16767-7. 29184159 PMC5705671

[ref50] Voyvodic P. L.; Bonnet J. Cell-Free Biosensors for Biomedical Applications. Curr. Opin. Biomed. Eng. 2020, 13, 9–15. 10.1016/j.cobme.2019.08.005.

[ref51] Thermostable T7 RNA Polymerase. TOYOBO USA. http://www.toyobousa.com/lifescience-thermostable-t7-rna-polymerase.html (accessed 2021-07-05).

[ref52] Sörensen M.; Mak T. N.; Hurwitz R.; Ogilvie L. A.; Mollenkopf H. J.; Meyer T. F.; Brüggemann H. Mutagenesis of *Propionibacterium acnes* and Analysis of Two CAMP Factor Knock-out Mutants. J. Microbiol. Methods 2010, 83 (2), 211–216. 10.1016/j.mimet.2010.09.008. 20850482

[ref53] Santos-Moreno J.; Schaerli Y. A Framework for the Modular and Combinatorial Assembly of Synthetic Gene Circuits. ACS Synth. Biol. 2019, 8 (7), 1691–1697. 10.1021/acssynbio.9b00174. 31185158

[ref54] Ko S.; Jeon H.; Yoon S.; Kyung M.; Yun H.; Na J. H.; Jung S. T. Discovery of Novel *Pseudomonas putida* Flavin-Binding Fluorescent Protein Variants with Significantly Improved Quantum Yield. J. Agric. Food Chem. 2020, 68 (21), 5873–5879. 10.1021/acs.jafc.0c00121. 32367716

