# Supplementary material for: Establishing a Cell-Free Transcription–Translation Platform for Cutibacterium acnes to Prototype Engineered Metabolic and Synthetic Biology
Source: ACS Biomater Sci Eng. 2021 Dec 31;9(9):5101–10. doi: 10.1021/acsbiomaterials.1c00894 (PMC10498419; doi:10.1021/acsbiomaterials.1c00894)
Supplement: Supplementary file 1 — ab1c00894_si_001.pdf [file ab1c00894_si_001.pdf]

Supplementary Information for:

Establishing a cell-free transcription-translation platform for *Cutibacterium acnes* to prototype engineered metabolic and synthetic biology.

María-José Fábrega[1], Nastassia Knödlseder[1], Guillermo Nevot[1], Marta Sanvicente[1], Lorena Toloza[1], Javier Santos-Moreno[1] and Marc Güell[1*].

[1] Department of Experimental and Health Sciences, Pompeu Fabra University, Barcelona, Spain

* Correspondence should be addressed to Prof. Marc Güell Cargol, Department of Experimental and Health Sciences, University Pompeu Fabra, Dr. Aiguader 88, 00803 Barcelona, Spain. Office: +34933160929. E-mail: marc.guell@upf.edu. Web: https://www.upf.edu/web/synbio.

1. Optimized composition of *C. acnes* CFS

Component	Concentration (reaction)	Units
**Nucleotides Mix**	1.2	mM
**Folinic acid**	0.069	mM
**E. coli tRNA**	0.17	mg/ml
**Aminoacids**	1	mM
**NAD**	0.33	mM
**CoA**	0.27	mM
**Spermidine**	1.5	mM
**Putrescine**	1	mM
**Sodium oxalate**	4	mg/ml
**Magnesium glutamate**	40	mM
**Potassium glutamate**	150	mM
**HEPES (pH 8)**	57	mM
**3-PGA**	30	mM
**PEG 6K**	1	% v/w
**T7 RNA polymerase**	16	µg/ml
**GamS nuclease inhibitor**	1,5	µg/ml
**Proteinase inhibitor**	1	X
**Lysate**	12	mg/ml
**DNA**	40	nM
**Water**	Up to 50 µl	

Table S1. Optimized composition of the *C. acnes* CFS reaction.

2. Detailed sequences for primers and constructs

NAME	SEQUENCE (5' TO 3')
GN_225	CAGCCTGCGGTCCGGACGGGCTTTGGCTTG
GN_226	GGGCGTCCCAGCGAGCTTTCCAGGATGAACCGAC
PR-UNA	ATAGGATAGATTCTGGAAACTTTACCGTCCGAGCTCCAGCCTGCGGTCCGG
PR-D17	TTCAACAGTATTGGGTCTGATAAGCACTACCGGGCGTCCCAGCGA
PR-U17	GTAGTGCTTATCAGACCCAATACTGTTGAACAGCCTGCGGTCCGG
PR-DNA	TAACGGAGAAGCCCTATCAACTGTTTATTGCTCGAGCGGGCGTCCCAGCGA
T7-Fluc RV	TCATGCTCTTCCCATTTACACGTGATGGTGGTGGTGGTGTAACTTACTTTTACCACCCTTCTTAGC
T7-Fluc FW	TAATACGACTCACTATAGGGTCCACTTATAAATATAGAGGAGGTTTGATATGGAGGATGCAAAGA ATATTAAGAAAG
pNM3_qPCR_fwd	ATGGCTAACACCGTAAACGTTC
pNM2_qPCR_fwd	ATGGCCAAGAAAGAGGGAGC
pNM1_qPCR_fwd	ATGGCTGTTTACACCCTTCC
pNM_qPCR_rev	TCAGTGGTGATGGTGATGATG

468	CCGGCCGCCATGGCAAACGTTCTGGCCGCAGATGG
469	CCTTCTGGTACCCTGTTTCCTCCAGATCGATCAG
470	TCGGGCTGGTACCCGATTCATGAGTGAACTCGTCC
471	AATTCACTAGTGCCAGCTGCTTGTCAGTCGAGA
499	GCCATCGAAGACGAACTTGAAG
500	ATCGGTGATGAGACCGTCCTT
308	ATTGTCCAGCCCACAACAGCAT
338	GTGCGAGTTCGACCTTCTGG

Table S2. Primers used for the C. acnes genetic engineering constructs and qPCR.

pGGA sequence:

CGAAAAATCAATAATCAGACAACAAGATGTGCGAACTCGATATTTTACACGACTCTCTTTACCAATTCTGCCCCGAATTACACTTAAAACGACTCAACAGCTTAACGTTGGCTTGCCACGCATTA
CTTGACTGTAAAACTCTCACTCTTACCGAACTTGGCCGTAACCTGCCAACCAAAGCGAGAACAAAACATAACATCAAACGAATCGACCGATTGTTAGGTAATCGTCACCTGCAGGAAGGTTTA
AACGCATTTAGGTGACACTATAGAAGTGTGTATCGCTCGAGGGATCCGAATTCGAAGACTTGGTACGGAGCGAGACCGCTTTCCAGATCTGATAACTTGTGGTCTCACCATTCCTGTAGTCTTC
TTAATTAAGACGTCAGAATTCTCGAGGCGGCCGCATGTGCGTCTCCCTATAGTGAGTCGTATTAATTTCGCGGGCGGAACCCCTATTTGTTTATTTTTCTAAATACATTCAAATATGTATCCGCT
CATGAGTAGCACCAGGCGTTTAAGGGCACCAATAACTGCCTTAAAAAAATTACGCCCCGCCCTGCCACTCATCGCAGTACTGTTGTAATTCATTAAGCATTCTGCCGACATGGAAGCCATCACA
AACGGCATGATGAACCTGAATCGCCAGCGGCATCAGCACCTTGTCGCCTTGCGTATAATATTTGCCCATGGTGAAAACGGGGGCGAAGAAGTTGTCCATATTGGCCACGTTTAAATCAAAACT
GGTGAAACTCACCCAGGGATTGGCTGAGACGAAAAACATATTCTCAATAAACCCTTTAGGGAAATAGGCCAGGTTTTCACCGTAACACGCCACATCTTGCGAATATATGTGTAGAAACTGCCG
GAAATCGTCGTGGTATTCACTCCAGAGCGATGAAAACGTTTCAGTTTGCTCATGGAAAACGGTGTAACAAGGGTGAACACTATCCCATATCACCAGCTCACCGTCTTTCATTGCCATACGGAAT
TCCGGATGAGCATTCATCAGGCGGGCAAGAATGTGAATAAAGGCCGGATAAAACTTGTGCTTATTTTTCTTTACGGTCTTTAAAAAGGCCGTAATATCCAGCTGAACGGTCTGGTTATAGGTA
CATTGAGCAACTGACTGAAATGCCTCAAAATGTTCTTTACGATGCCATTGGGATATATCAACGGTGGTATATCCAGTGATTTTTTTCTCCATTTTAGCTTCCTTAGCTCCTGAAAATCTCGATAAC
TCAAAAAATACGCCCGGTAGTGATCTTATTTCATTATGGTGAAAGTTGGAACCTCTTACGTGCCGATCAACGTCTCATTTTCGCCAAAAGTTGTCATGACCAAAATCCCTTAACGTGAGTTTTCG
TTCCACTGAGCGTCAGACCCCGTAGAAAAGATCAAAGGATCTTCTTGAGATCCTTTTTTTCTGCGCGTAATCTGCTGCTTGCAAACAAAAAAACCACCGCTACCAGCGGTGGTTTGTTTGCCGG
ATCAAGAGCTACCAACTCTTTTTCCGAAGGTAACTGGCTTCAGCAGAGCGCAGATACCAAATACTGTTCTTCTAGTGTAGCCGTAGTTAGGCCACCACTTCAAGAACTCTGTAGCACCGCCTAC
ATACCTCGCTCTGCTAATCCTGTTACCAGTGGCTGCTGCCAGTGGCGATAAGTCGTGTCTTACCGGGTTGGACTCAAGACGATAGTTACCGGATAAGGCGCAGCGGTCGGGCTGAACGGGGG
GTTCGTGCACACAGCCCAGCTTGGAGCGAACGACCTACACCGAACTGAGATACCTACAGCGTGAGCTATGAGAAAGCGCCACGCTTCCCGAAGGGAGAAAGGCGGACAGGTATCCGGTAAG
CGGCAGGGTCGGAACAGGAGAGCGCACGAGGGAGCTTCCAGGGGGAAACGCCTGGTATCTTTATAGTCCTGTCGGGTTTCGCCACCTCTGACTTGAGCGTCGATTTTTGTGATGCTCGTCAG
GGGGGCGGAGCCTATGGAAAAACGCCAGCAATGCGGCCTTTTTACGGTTCCTGGCCTTTTGCTGGCCTTTTGCTCACATGTTCTTTCCTGCGTTATCCCCTGATTCTGTGGATAACCGTATTACC
GCCTTTGAGTGAGCTGATACCGCTCGCCGCAGCCGAACGACCGAGCGCAGCGAGTCAGTGAGCGAGGAAGC

T7-fyrefly luciferase *C. acnes* CO:

TCATGCTCTTCCAGCTAATACGACTCACTATAGGGTCCACTTATAAATATAGAGGAGGTTTGATATGGAGGATGCAAAGAATATTAAGAAAGGTCCAGCTCCTTTCTACCCTTTAGAAGACGGA
ACTGCTGGTGAGCAATTACACAAGGCAATGAAGAGGTATGCATTAGTACCAGGAACTATAGCTTTTACTGATGCTCATATTGAAGTAAATATAACATATGCAGAATACTTTGAGATGTCTGTG
AGGCTTGCAGAAGCAATGAAAAGATATGGATTAAATACTAACCACAGGATAGTGGTTTGTTCTGAAAACAGCTTACAATTCTTCATGCCAGTTCTTGGAGCATTATTCATTGGAGTTGCTGTGG
CTCCAGCAAATGACATTTACAACGAGAGGGAGTTGTTAAATTCAATGAATATTAGTCAACCTACTGTAGTGTTCGTTTCTAAGAAGGGACTTCAGAAAATTCTAAACGTGCAAAAAAAGCTAC
CAATTATTCAAAAGATAATAATTATGGACTCAAAAACTGATTACCAAGGATTCCAGAGCATGTATACTTTTGTTACATCTCATCTACCACCAGGTTTTAATGAGTATGATTTCGTGCCAGAAAGC
TTTGACAGAGATAAGACAATAGCTTTGATTATGAACAGCTCAGGATCTACAGGACTACCTAAGGGTGTGGCTCTACCTCATAGGACTGCTTGCGTTAGGTTTAGTCATGCAAGGGACCCTATA
TTTGGAAATCAAATTATTCCTGATACTGCAATACTATCAGTTGTACCATTTCATCACGGATTCGGTATGTTCACAACATTGGGATATCTTATATGCGGTTTTAGAGTGGTACTTATGTATAGGTTC
GAGGAAGAACTTTTTTTAAGGAGTCTACAAGACTACAAAATACAATCAGCATTGTTAGTGCCAACATTATTTAGTTTTTTCGCTAAAAGCACACTTATAGATAAATACGACTTGTCTAACCTACA
CGAAATAGCAAGCGGTGGAGCTCCTTTATCTAAAGAGGTAGGAGAAGCTGTTGCAAAAAGATTCCACTTACCTGGTATAAGACAGGGTTATGGATTGACAGAAACAACATCAGCAATTTTGA
TTACTCCAGAGGGAGATGACAAGCCAGGTGCTGTAGGAAAGGTGGTACCATTCTTTGAAGCTAAAGTAGTAGACTTGGACACAGGAAAAACTCTAGGTGTGAATCAGAGAGGAGAACTTTG
CGTAAGGGGACCAATGATAATGTCAGGTTATGTAAATAATCCAGAGGCAACAAATGCACTTATAGATAAAGATGGTTGGTTGCACAGCGGAGATATAGCTTACTGGGATGAGGACGAACATT
TTTTTATTGTGGACAGGCTTAAAAGTTTGATTAAATACAAAGGTTACCAGGTGGCACCAGCTGAGCTTGAATCTATATTGCTTCAACACCCAAATATTTTTGATGCTGGTGTAGCAGGTCTTCCT
GATGATGATGCAGGAGAGCTTCCAGCTGCTGTAGTAGTATTAGAGCACGGAAAGACAATGACAGAAAAGGAAATAGTGGACTACGTTGCATCTCAGGTTACAACTGCAAAGAAGCTAAGGG
GAGGTGTTGTTTTTGTAGACGAAGTTCCTAAAGGATTGACAGGAAAGTTGGACGCTAGGAAGATTAGAGAGATTCTAATAAAAGCTAAGAAGGGTGGTAAAAGTAAGTTACACCACCACCA
CCATCACGTGTAAATGGGAAGAGCATGA

pNA-14

TTTTCTACTGAACCGCTCTAGATTTCAGTGCAATTTATCTCTTCAAATGTAGCACCTGAAGTCAGCCCCATACGATATAAGTTGTAATTCTCATGTTAGTCATGCCCCGCGCCCACCGGAAGGAG
CTGACTGGGTTGCTCCTAGGATGCAGTGACTCGCACATTTCCCCGAAAAGTGCCACCTGAATTCATAGGATAGATTCTGGAAACTTTACCGTCCGAGCTCCAGCCTGCGGTCCGGACGGGCTT
TGGCTTGAACCATCGGAACGCGGGCTTTCTGTCACTGATCTGGACGGTAGTCACGATTGTGCAGGCAGTTCGAGGGTTCAAAGGGCAAGACTGAGACACAGGTATCGCGGCTGCGGGCGGC
ACCTATGACCACGGTCTACCTCAAGGCGAGTTCGGTTGGTTAGGCGTTGTCGTCGGTTCATCCTGGAAAGCTCGCTGGGACGCCCGGTAGTGCTTATCAGACCCAATACTGTTGAACAGCCTG
CGGTCCGGTCCACTTATAAATATAGAGGAGGTTTGATATGGATTATAAAGATCACGACGGAGATTACAAAGACCACGATATAGATTACAAAGACGATGACGATAAAATTAATGCAAAACTATT
ACAACTTATGGTAGAATACTCAAACGATGGAATAGTAGTAGCAGAGCCAGAGGGAAACGAATCGATACTTATATATGTTAACCCCGCATTTGAAAGATTAACAGGATATTGTGCAGACGACA
TACTTTACCAAGACGGGAGATTCTTACAAGGCGAAGATCATGACCAACCAGGAATAGCAATTATACGGGAAGCCATACGTGAAGGGAGACCATGCTGCCAAGTACTTAGAAATTATCGAAAA
GACGGCTCGCTTTTTTGGAACGAATTGTCCATCACCCCAGTACATAACGAAGCAGACAGACTAACATACTTCATAGGAACTCAGAGAGATGTTACAGCCCAAGTGTTCGCGGAAGAAAGAAT
GAGAGAGTTAGAAGCGGAAGTTGCAGAATTACGGAGACAATAATCGCTGGGACGCCCGCTCGAGCAATAAACAGTTGATAGGGCTTCTCCGTTACCATGGTTCAGCCAAAAAACTTAAGACC
GCCGGTCTTGTCCACTACCTTGCAGTAATGCGGTGGACAGGATCGGCGGTTTTCTTTTCTCTTCTCAATTCTTCTGACCTGTAACGAATAATAGATAGTAAAGTAGTCTCCGATTGAGTTTTCTC
TGCCGAGTCCCACCCAGTTCTGTGATTTCAGTAAGTTGGTAATTGATACACTGTTGCGAGAACTGGTGCCTGGTAGTAGATAGGTTGTTATTGAGTAAGAAGGTAAAGTGAACGAAATCCCTG
AAACTGAGACTGTAGAAAATAAGCTTTGCTGCCACCGCTGAGCAATAACTAGCATAACCCCTTGGGGCCTCTAAACGGGTCTTGAGGGGTTTTTTGCCAGGCATCAAATAAAACGAAAGGCTC
AGTCGGAAGACTGGGCCTTTCGTTTTATCTGTTGTTTGTCGGTGAACGCTCTCCTGAGTAGGACAAATCCGCCGGGAGCGGATTTGAACGTTGTGAAGCAACGGCCCGGAGGGTGGCGGGCA
GGACGCCCGCCATAAACTGCCAGGCATCAAACTAAGCAGAAGGCCATCCTGACGGATGGCCTTTTTGCGTTTCAGATCTACCGGTAAACCAGCAATAGACATAAGCGGCTATTTAACGACCCT
GCCCTGAACCGACGACCGGGTCATCGTGGCCGGATCTTGCGGCCCCTCGGCTTGAACGAATTGTTAGACATTATTTGCCGACTACCTTGGTGATCTCGCCTTTCACGTAGTGGACAAATTCTTC
CAACTGATCTGCGCGCGAGGCCAAGCGATCTTCTTCTTGTCCAAGATAAGCCTGTCTAGCTTCAAGTATGACGGGCTGATACTGGGCCGGCAGGCGCTCCATTGCCCAGTCGGCAGCGACATC
CTTCGGCGCGATTTTGCCGGTTACTGCGCTGTACCAAATGCGGGACAACGTAAGCACTACATTTCGCTCATCGCCAGCCCAGTCGGGCGGCGAGTTCCATAGCGTTAAGGTTTCATTTAGCGC
CTCAAATAGATCCTGTTCAGGAACCGGATCAAAGAGTTCCTCCGCCGCTGGACCTACCAAGGCAACGCTATGTTCTCTTGCTTTTGTCAGCAAGATAGCCAGATCAATGTCGATCGTGGCTGGC
TCGAAGATACCTGCAAGAATGTCATTGCGCTGCCATTCTCCAAATTGCAGTTCGCGCTTAGCTGGATAACGCCACGGAATGATGTCGTCGTGCACAACAATGGTGACTTCTACAGCGCGGAGA
ATCTCGCTCTCTCCAGGGGAAGCCGAAGTTTCCAAAAGGTCGTTGATCAAAGCTCGCCGCGTTGTTTCATCAAGCCTTACGGTCACCGTAACCAGCAAATCAATATCACTGTGTGGCTTCAGGC
CGCCATCCACTGCGGAGCCGTACAAATGTACGGCCAGCAACGTCGGTTCGAGATGGCGCTCGATGACGCCAACTACCTCTGATAGTTGAGTCGATACTTCGGCGATCACCGCTTCCCTCATAC
TCTTCCTTTTTCAATATTATTGAAGCATTTATCAGGGTTATTGTCTCATGAGCGGATACATATTTGAATGTATTTAGAAAAATAAACAAATAGCTAGCTCACTCGGTCGCTACGCTCCGGGCGTG
AGACTGCGGCGGGCGCTGCGGACACATACAAAGTTACCCACAGATTCCGTGGATAAGCAGGGGACTAACATGTGAGGCAAAACAGCAGGGCCGCGCCGGTGGCGTTTTTCCATAGGCTCCG
CCCTCCTGCCAGAGTTCACATAAACAGACGCTTTTCCGGTGCATCTGTGGGAGCCGTGAGGCTCAACCATGAATCTGACAGTACGGGCGAAACCCGACAGGACTTAAAGATCCCCACCGTTTC
CGGCGGGTCGCTCCCTCTTGCGCTCTCCTGTTCCGACCCTGCCGTTTACCGGATACCTGTTCCGCCTTTCTCCCTTACGGGAAGTGTGGCGCTTTCTCATAGCTCACACACTGGTATCTCGGCTC
GGTGTAGGTCGTTCGCTCCAAGCTGGGCTGTAAGCAAGAACTCCCCGTTCAGCCCGACTGCTGCGCCTTATCCGGTAACTGTTCACTTGAGTCCAACCCGGAAAAGCACGGTAAAACGCCACT
GGCAGCAGCCATTGGTAACTGGGAGTTCGCAGAGGATTTGTTTAGCTAAACACGCGGTTGCTCTTGAAGTGTGCGCCAAAGTCCGGCTACACTGGAAGGACAGATTTGGTTGCTGTGCTCTG
CGAAAGCCAGTTACCACGGTTAAGCAGTTCCCCAACTGACTTAACCTTCGATCAAACCACCTCCCCAGGTGGTTTTTTCGTTTACAGGGCAAAAGATTACGCGCAGAAAAAAAGGATCTCAAG
AAGATCCTTTGATC

pNM1

AGACTGGATGGAGGCGGATAAAGTTGCAGGACCACTTCTGCGCTCGGCCCTTCCGGCTGGCTGGTTTATTGCTGATAAATCTGGAGCCGGTGAGCGTGGGTCTCGCGGTATCATTGCAGCAC
TGGGGCCAGATGGTAAGCCCTCCCGTATCGTAGTTATCTACACGACGGGGAGTCAGGCAACTATGGATGAACGAAATAGACAGATCGCTGAGATAGGTGCCTCACTGATTAAGCATTGGTAA
CTGTCAGACCAAGTTTACTCATATATACTTTAGATTGATTTAAAACTTCATTTTTAATTTAAAAGGATCTAGGTGAAGATCCTTTTTGATAATCTCATGACCAAAATCCCTTAACGTGAGTTTTCG
TTCCACTGAGCGTCAGACCCCGTAGAAAAGATCAAAGGATCTTCTTGAGATCCTTTTTTTCTGCGCGTAATCTGCTGCTTGCAAACAAAAAAACCACCGCTACCAGCGGTGGTTTGTTTGCCGG
ATCAAGAGCTACCAACTCTTTTTCCGAAGGTAACTGGCTTCAGCAGAGCGCAGATACCAAATACTGTTCTTCTAGTGTAGCCGTAGTTAGGCCACCACTTCAAGAACTCTGTAGCACCGCCTAC
ATACCTCGCTCTGCTAATCCTGTTACCAGTGGCTGCTGCCAGTGGCGATAAGTCGTGTCTTACCGGGTTGGACTCAAGACGATAGTTACCGGATAAGGCGCAGCGGTCGGGCTGAACGGGGG
GTTCGTGCACACAGCCCAGCTTGGAGCGAACGACCTACACCGAACTGAGATACCTACAGCGTGAGCTATGAGAAAGCGCCACGCTTCCCGAAGGGAGAAAGGCGGACAGGTATCCGGTAAG
CGGCAGGGTCGGAACAGGAGAGCGCACGAGGGAGCTTCCAGGGGGAAACGCCTGGTATCTTTATAGTCCTGTCGGGTTTCGCCACCTCTGACTTGAGCGTCGATTTTTGTGATGCTCGTCAG

GGGGGCGGAGCCTATGGAAAAACGCCAGCAACGCGGCCTTTTTACGGTTCCTGGCCTTTTGCTGGCCTTTTGCTCACATGTTCTTTCCTGCGTTATCCCCTGATTCTGTGGATAACCGTATTACC
GCCTTTGAGTGAGCTGATACCGCTCGCCGCAGCCGAACGACCGAGCGCAGCGAGTCAGTGAGCGAGGAAGCGCTACGGCCGAATAGGACCAGTTCCTCCACAGCGCGTGAAAAATCTCGCA
AACACGGGAATGATGGAAACAGGTTCGGTGTTGTACCACGTAAGCGGCTCTGCCAAGGGCCACGTCATGACGGACGTCGTGACGTATCTCGTTCCACAGGAGGAAGAATGGCTGTTTACACC
CTTCCCGATCTCGACTACGACTACGGAGCACTGGAGCCCCACATCTCGGGCAAGATCATGGAACTGCACCATGACAAGCACCACAACACCTACGTTCAGGGTGCCAACACCGCCCTGGAGAA
GCTGGCCGAGGCCCGCGAGAAGGGCGACTTCGGAACCATCAACAAACTCGAAAAGGACCTGGCCTTTAACCTCGGCGGCCACATCAACCACTCCGTGTTCTGGAAGAACATGTCCCCTCATG
GGGGCGGTCGTCCGGAGGGCAACGAACTCGCTGCTGCGATTGACGAGTTCTTCGGTTCCTTTGACAGCTTCAAAAAGCAGTTTGAGGAAACCGCTAAGGGCGTTCAGGGCTCCGGCTGGGG
CATGCTCGTGTGGGACGTGATGGGTCAGCGCCTCAACACCATGCAGCTGTTTGACCACCAGGGCAATCTGCCGTCAACCCAGATCCCGGTCGTCCAGCTCGACATGTGGGAACACGCTTATTA
CCTGCAGTACCAGAACGTCAAGGCCGACTACGTTACCGCCTGGTGGAACGTCGTTAGCTGGACCGACGCCGAGCAGCGGTTCGTCAAGGCCCGTTCGATCACCGGTTTGTTCCATCATCACCA
TCACCACTGAATGGGCGAGCTCGAATTCACTGGCCGTCGTTTTACAACGTCGTGACTGGGAAAACCCTGGCGTTACCCAACTTAATCGCCTTGCAGCACATCCCCCTTTCGCCAGCTGGCGTAA
TAGCGAAGAGGCCCGCACCGATCGCCCTTCCCAACAGTTGCGCAGCCTGAATGGCGAATGGCGCCTGATGCGGTATTTTCTCCTTACGCATCTGTGCGGTATTTCACACCGCATATGGTGCAC
TCTCAGTACAATCTGCTCTGATGCCGCATAGTTAAGCCAGCCCCGACACCCGCCAACACCCGCTGACGCGCCCTGACGGGCTTGTCTGCTCCCGGCATCCGCTTACAGACAAGCTGTGACCGTC
TCCGGGAGCTGCATGTGTCAGAGGTTTTCACCGTCATCACCGAAACGCGCGAGACGAAAGGGCCTCGTGATACGCCTATTTTTATAGGTTAATGTCATGATAATAATGGTTTCTTAGACGTCA
GGTGGCACTTTTCGGGGAAATGTGCGCGGAACCCCTATTTGTTTATTTTTCTAAATACATTCAAATATGTATCCGCTCATGAGACAATAACCCTGATAAATGCTTCAATAATATTGAAAAAGGA
AGAGTATGAGTATTCAACATTTCCGTGTCGCCCTTATTCCCTTTTTTGCGGCATTTTGCCTTCCTGTTTTTGCTCACCCAGAAACGCTGGTGAAAGTAAAAGATGCTGAAGATCAGTTGGGTGCA
CGAGTGGGTTACATCGAACTGGATCTCAACAGCGGTAAGATCCTTGAGAGTTTTCGCCCCGAAGAACGTTTTCCAATGATGAGCACTTTTAAAGTTCTGCTATGTGGCGCGGTATTATCCCGTA
TTGACGCCGGGCAAGAGCAACTCGGTCGCCGCATACACTATTCTCAGAATGACTTGGTTGAGTACTCACCAGTCACAGAAAAGCATCTTACGGATGGCATGACAGTAAGAGAATTATGCAGT
GCTGCCATAACCATGAGTGATAACACTGCGGCCAACTTACTTCTGACAACGATCGGAGGACCGAAGGAGCTAACCGCTTTTTTGCACAACATGGGGGATCATGTAACTCGCCTTGATCGTTGG
GAACCGGAGCTGAATGAAGCCATACCAAACGACGAGCGTGACACCACGATGCCTGTAGCAATGGCAACAACGTTGCGCAAACTATTAACTGGCGAACTACTTACTCTAGCTTCCCGGCAACA
ATTAAT

pNM2

ACCGTCATCACCGAAACGCGCGAGACGAAAGGGCCTCGTGATACGCCTATTTTTATAGGTTAATGTCATGATAATAATGGTTTCTTAGACGTCAGGTGGCACTTTTCGGGGAAATGTGCGCGG
AACCCCTATTTGTTTATTTTTCTAAATACATTCAAATATGTATCCGCTCATGAGACAATAACCCTGATAAATGCTTCAATAATATTGAAAAAGGAAGAGTATGAGTATTCAACATTTCCGTGTCGC
CCTTATTCCCTTTTTTGCGGCATTTTGCCTTCCTGTTTTTGCTCACCCAGAAACGCTGGTGAAAGTAAAAGATGCTGAAGATCAGTTGGGTGCACGAGTGGGTTACATCGAACTGGATCTCAAC
AGCGGTAAGATCCTTGAGAGTTTTCGCCCCGAAGAACGTTTTCCAATGATGAGCACTTTTAAAGTTCTGCTATGTGGCGCGGTATTATCCCGTATTGACGCCGGGCAAGAGCAACTCGGTCGC
CGCATACACTATTCTCAGAATGACTTGGTTGAGTACTCACCAGTCACAGAAAAGCATCTTACGGATGGCATGACAGTAAGAGAATTATGCAGTGCTGCCATAACCATGAGTGATAACACTGCG
GCCAACTTACTTCTGACAACGATCGGAGGACCGAAGGAGCTAACCGCTTTTTTGCACAACATGGGGGATCATGTAACTCGCCTTGATCGTTGGGAACCGGAGCTGAATGAAGCCATACCAAA
CGACGAGCGTGACACCACGATGCCTGTAGCAATGGCAACAACGTTGCGCAAACTATTAACTGGCGAACTACTTACTCTAGCTTCCCGGCAACAATTAATAGACTGGATGGAGGCGGATAAAG
TTGCAGGACCACTTCTGCGCTCGGCCCTTCCGGCTGGCTGGTTTATTGCTGATAAATCTGGAGCCGGTGAGCGTGGGTCTCGCGGTATCATTGCAGCACTGGGGCCAGATGGTAAGCCCTCCC
GTATCGTAGTTATCTACACGACGGGGAGTCAGGCAACTATGGATGAACGAAATAGACAGATCGCTGAGATAGGTGCCTCACTGATTAAGCATTGGTAACTGTCAGACCAAGTTTACTCATATA
TACTTTAGATTGATTTAAAACTTCATTTTTAATTTAAAAGGATCTAGGTGAAGATCCTTTTTGATAATCTCATGACCAAAATCCCTTAACGTGAGTTTTCGTTCCACTGAGCGTCAGACCCCGTAG
AAAAGATCAAAGGATCTTCTTGAGATCCTTTTTTTCTGCGCGTAATCTGCTGCTTGCAAACAAAAAAACCACCGCTACCAGCGGTGGTTTGTTTGCCGGATCAAGAGCTACCAACTCTTTTTCCG
AAGGTAACTGGCTTCAGCAGAGCGCAGATACCAAATACTGTTCTTCTAGTGTAGCCGTAGTTAGGCCACCACTTCAAGAACTCTGTAGCACCGCCTACATACCTCGCTCTGCTAATCCTGTTAC
CAGTGGCTGCTGCCAGTGGCGATAAGTCGTGTCTTACCGGGTTGGACTCAAGACGATAGTTACCGGATAAGGCGCAGCGGTCGGGCTGAACGGGGGGTTCGTGCACACAGCCCAGCTTGGA
GCGAACGACCTACACCGAACTGAGATACCTACAGCGTGAGCTATGAGAAAGCGCCACGCTTCCCGAAGGGAGAAAGGCGGACAGGTATCCGGTAAGCGGCAGGGTCGGAACAGGAGAGC
GCACGAGGGAGCTTCCAGGGGGAAACGCCTGGTATCTTTATAGTCCTGTCGGGTTTCGCCACCTCTGACTTGAGCGTCGATTTTTGTGATGCTCGTCAGGGGGGCGGAGCCTATGGAAAAAC
GCCAGCAACGCGGCCTTTTTACGGTTCCTGGCCTTTTGCTGGCCTTTTGCTCACATGTTCTTTCCTGCGTTATCCCCTGATTCTGTGGATAACCGTATTACCGCCTTTGAGTGAGCTGATACCGCT
CGCCGCAGCCGAACGACCGAGCGCAGCGAGTCAGTGAGCGAGGAAGCGGTTGATCTGTAGTGCTGCCGGGGCCGTGGGTTCCGGCAGCACTGTGGACTACGGGCGGGACGCGTGCCCTCT
GTGTGAGTTACTGCCGGTGCCCTTTAGCTCCTTTCAATTTGGGGAGACGACGTTGAGAAGGTATCGTTAACTGCTGGCTTCGTGTGTGCGGAGCCACAACCACGCCCGCAGGGGGTGTGGGT
AGCGGACAGATGGGACGTAATGGCCAAGAAAGAGGGAGCACTCGAACTGGAGGGAACTGTCGTTGAAGCTTTGCCCAACGCCATGTTCCGCGTCGAACTCAAGAATGGGCACCGCGTTCTT
GCCACGATCAGCGGCAAGATGCGTCAACACTACATCCGAATCCTGCCGTCGGACCGTGTCGTTGTTGAGCTGTCGCCCTACGATCTCACCCGCGGCCGGATCGTCTACCGCCACAAGCATCAT
CACCATCACCACTGAATGGGCGAGCTCGAATTCACTGGCCGTCGTTTTACAACGTCGTGACTGGGAAAACCCTGGCGTTACCCAACTTAATCGCCTTGCAGCACATCCCCCTTTCGCCAGCTGG
CGTAATAGCGAAGAGGCCCGCACCGATCGCCCTTCCCAACAGTTGCGCAGCCTGAATGGCGAATGGCGCCTGATGCGGTATTTTCTCCTTACGCATCTGTGCGGTATTTCACACCGCATATGG
TGCACTCTCAGTACAATCTGCTCTGATGCCGCATAGTTAAGCCAGCCCCGACACCCGCCAACACCCGCTGACGCGCCCTGACGGGCTTGTCTGCTCCCGGCATCCGCTTACAGACAAGCTGTGA
CCGTCTCCGGGAGCTGCATGTGTCAGAGGTTTT

pNM3

AACTGGCGAACTACTTACTCTAGCTTCCCGGCAACAATTAATAGACTGGATGGAGGCGGATAAAGTTGCAGGACCACTTCTGCGCTCGGCCCTTCCGGCTGGCTGGTTTATTGCTGATAAATC
TGGAGCCGGTGAGCGTGGGTCTCGCGGTATCATTGCAGCACTGGGGCCAGATGGTAAGCCCTCCCGTATCGTAGTTATCTACACGACGGGGAGTCAGGCAACTATGGATGAACGAAATAGA
CAGATCGCTGAGATAGGTGCCTCACTGATTAAGCATTGGTAACTGTCAGACCAAGTTTACTCATATATACTTTAGATTGATTTAAAACTTCATTTTTAATTTAAAAGGATCTAGGTGAAGATCCT
TTTTGATAATCTCATGACCAAAATCCCTTAACGTGAGTTTTCGTTCCACTGAGCGTCAGACCCCGTAGAAAAGATCAAAGGATCTTCTTGAGATCCTTTTTTTCTGCGCGTAATCTGCTGCTTGCA
AACAAAAAAACCACCGCTACCAGCGGTGGTTTGTTTGCCGGATCAAGAGCTACCAACTCTTTTTCCGAAGGTAACTGGCTTCAGCAGAGCGCAGATACCAAATACTGTTCTTCTAGTGTAGCC
GTAGTTAGGCCACCACTTCAAGAACTCTGTAGCACCGCCTACATACCTCGCTCTGCTAATCCTGTTACCAGTGGCTGCTGCCAGTGGCGATAAGTCGTGTCTTACCGGGTTGGACTCAAGACGA
TAGTTACCGGATAAGGCGCAGCGGTCGGGCTGAACGGGGGGTTCGTGCACACAGCCCAGCTTGGAGCGAACGACCTACACCGAACTGAGATACCTACAGCGTGAGCTATGAGAAAGCGCC
ACGCTTCCCGAAGGGAGAAAGGCGGACAGGTATCCGGTAAGCGGCAGGGTCGGAACAGGAGAGCGCACGAGGGAGCTTCCAGGGGGAAACGCCTGGTATCTTTATAGTCCTGTCGGGTTT
CGCCACCTCTGACTTGAGCGTCGATTTTTGTGATGCTCGTCAGGGGGGCGGAGCCTATGGAAAAACGCCAGCAACGCGGCCTTTTTACGGTTCCTGGCCTTTTGCTGGCCTTTTGCTCACATGT
TCTTTCCTGCGTTATCCCCTGATTCTGTGGATAACCGTATTACCGCCTTTGAGTGAGCTGATACCGCTCGCCGCAGCCGAACGACCGAGCGCAGCGAGTCAGTGAGCGAGGAAGCAAGTCACA
TCATGCCCTATGAGGGGCATCTAGCCGACACAAAGAGCGACCAGCACTCGGCCGCCCACATTTTTCGTGTCTGGGATGGAATAACACACGTTCGGCCCGTGTTGCCCTATATGTACGCCGGTG
GTCGGCGCTTGGCCACCCCGAACGAAGGAGATGTTGCATGGCTAACACCGTAAACGTTCCGAATCCTGCCGGACAGACCACTACTAAGCTCGAAAACGCTGAATCCGGTTTCAAGGCCTCCG
AAACCCTTGCCGCGAACCTTCAAAAGGTTCTCGTTAACTTCATCGCCTTGGAGTTGGTCGGCAAACATGCCCACTGGAATATCGTCGGCCCCAACTTCCGTGACCTCCACCTCAACCTCGACGA
GGTCGTCGAAATTGCCCGTGAGGGCGCTGACGAGATCGCTGAGCGTATGCGCGCCCTTCACGCCTCCCCAGACGGTCGTCCCGCAGTCGTAGCTGCGAGCACCACCCTGCCGGAATCCCCTC
AGGGCGAGGTACTGACCCACGACGCCATCAAGGTCGTCACCGCTTCAATCGAGTCCGTGACCGGCACTATGCGTGAGGTCCACGATCAGGTTGACGAAGAGGACCCCACCTCCGCTGACATC
CTGCACGGCCTCATCCAGCAGCTTGAACAGCAGGCATGGTTCATCAGCGCTGAAACTCGCACTCCAACTGCTCACCATCATCACCATCACCACTGAATGGGCGAGCTCGAATTCACTGGCCGTC
GTTTTACAACGTCGTGACTGGGAAAACCCTGGCGTTACCCAACTTAATCGCCTTGCAGCACATCCCCCTTTCGCCAGCTGGCGTAATAGCGAAGAGGCCCGCACCGATCGCCCTTCCCAACAGT
TGCGCAGCCTGAATGGCGAATGGCGCCTGATGCGGTATTTTCTCCTTACGCATCTGTGCGGTATTTCACACCGCATATGGTGCACTCTCAGTACAATCTGCTCTGATGCCGCATAGTTAAGCCA
GCCCCGACACCCGCCAACACCCGCTGACGCGCCCTGACGGGCTTGTCTGCTCCCGGCATCCGCTTACAGACAAGCTGTGACCGTCTCCGGGAGCTGCATGTGTCAGAGGTTTTCACCGTCATC
ACCGAAACGCGCGAGACGAAAGGGCCTCGTGATACGCCTATTTTTATAGGTTAATGTCATGATAATAATGGTTTCTTAGACGTCAGGTGGCACTTTTCGGGGAAATGTGCGCGGAACCCCTAT
TTGTTTATTTTTCTAAATACATTCAAATATGTATCCGCTCATGAGACAATAACCCTGATAAATGCTTCAATAATATTGAAAAAGGAAGAGTATGAGTATTCAACATTTCCGTGTCGCCCTTATTCC
CTTTTTTGCGGCATTTTGCCTTCCTGTTTTTGCTCACCCAGAAACGCTGGTGAAAGTAAAAGATGCTGAAGATCAGTTGGGTGCACGAGTGGGTTACATCGAACTGGATCTCAACAGCGGTAA
GATCCTTGAGAGTTTTCGCCCCGAAGAACGTTTTCCAATGATGAGCACTTTTAAAGTTCTGCTATGTGGCGCGGTATTATCCCGTATTGACGCCGGGCAAGAGCAACTCGGTCGCCGCATACAC
TATTCTCAGAATGACTTGGTTGAGTACTCACCAGTCACAGAAAAGCATCTTACGGATGGCATGACAGTAAGAGAATTATGCAGTGCTGCCATAACCATGAGTGATAACACTGCGGCCAACTTA
CTTCTGACAACGATCGGAGGACCGAAGGAGCTAACCGCTTTTTTGCACAACATGGGGGATCATGTAACTCGCCTTGATCGTTGGGAACCGGAGCTGAATGAAGCCATACCAAACGACGAGCG
TGACACCACGATGCCTGTAGCAATGGCAACAACGTTGCGCAAACTATT

Table S3. Plasmids/Genes used in the C. acnes CFS reaction. In blue are highlighted the promoters, in green the RBS and in grey the reporter gene.

3. Contamination test for *C. acnes* cell lysates

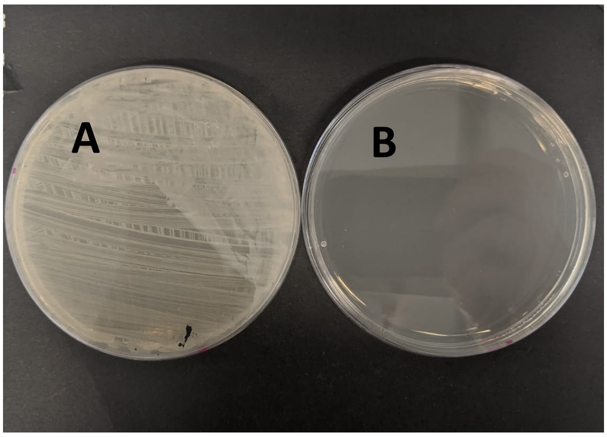

Figure S1. Contamination test of *C. acnes* cell lysate. After bacterial lysis process, 50 µl of clarified supernatants were seeded on Brucella agar plates and incubated in anaerobiosis using the GasPack System for 3-5 days. A) Example of a normal culture of C. acnes after 6 days growing. B) *C. acnes* lysate after 6 days of incubation.

4. *C. acnes* growth curve

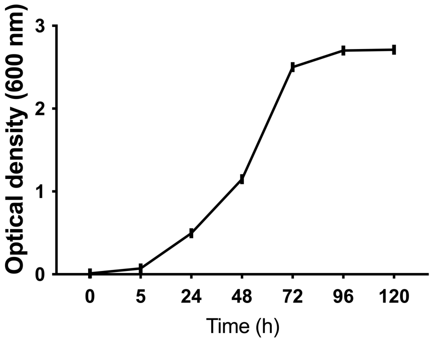

Figure S2. Growth curve of *C . acnes* restriction methylation deficient KPA strain.

5. Standard curve of reference for luciferase emission.

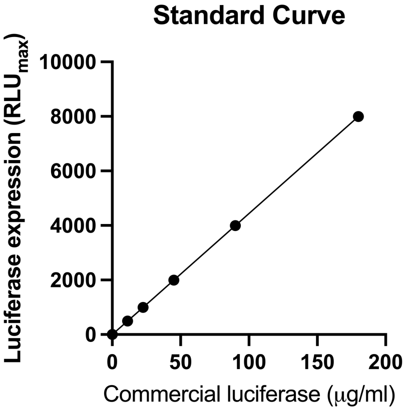

Figure S3. Standard curve for commercial luciferase. In our *C. acnes* CFS we got a maximum amount of 2600 RLUs which correspond to approximately 85 µg/ml of enzyme.

6. Optimization of additional *C. acnes* CFS parameters

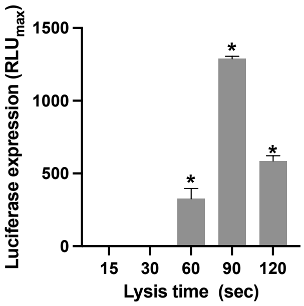

Figure S4. Luciferase expression using different *C. acnes* lysates. Using the Freezer Mill and keeping 5CPS, the C. acnes pellet was disrupted during different times (15, 30, 60, 90 and 120 seconds). After a 3h-CFS reaction samples were assayed for luminescence. Results are presented as mean ± standard deviation (SD) of triplicate measurements ($p < 0.05$) versus negative control.

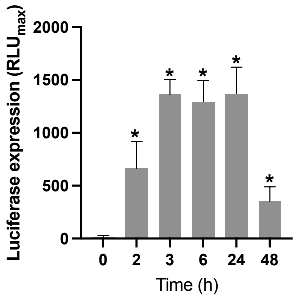

Figure S5. *C. acnes* CFS time course reaction. Different CFS reactions were run in parallel but at different time of incubation at 30°C. Results are presented as mean ± standard deviation (SD) of triplicate measurements ($p < 0.05$) versus negative control.

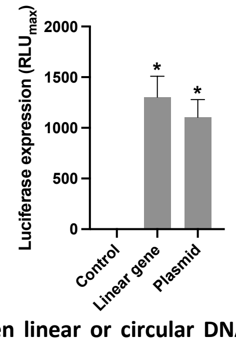

Figure S6. *C. acnes* CFS comparison between linear or circular DNA. Different 3h-CFS reactions were prepared containing the same buffer and lysate but differing in the structure of DNA (40 nM of linear or circular) but having both of them the same reporter T7-Fluc. For circular constructs the Topo Cloning system was used. Results are presented as mean ± standard deviation (SD) of triplicate measurements ($p < 0.05$) versus negative control.

7. Transcriptomic analysis for constitutive promoters identification

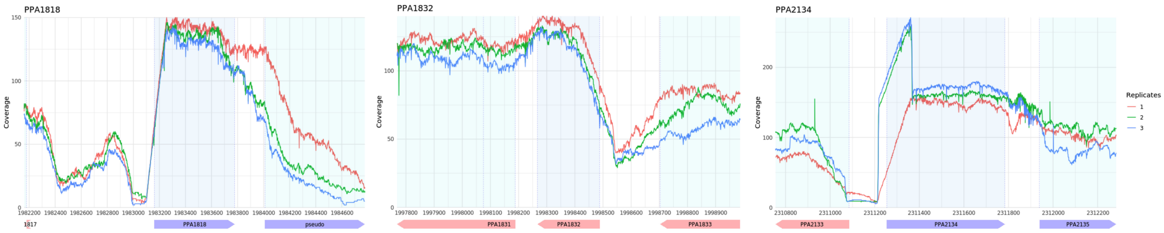

Figure S7. ***C. acnes*** **transcriptomic analysis for screening of putative promoters**. Promoters identified in the C. acnes CFS RNAseq as highly expressed (PPA1818, PPA1832 and PPA2134) were also detected in the total genomic C. acnes transcriptomic analysis as one of the most expressed. The panels show, from top to bottom, the coverage, reads, and genomic annotations from three samples

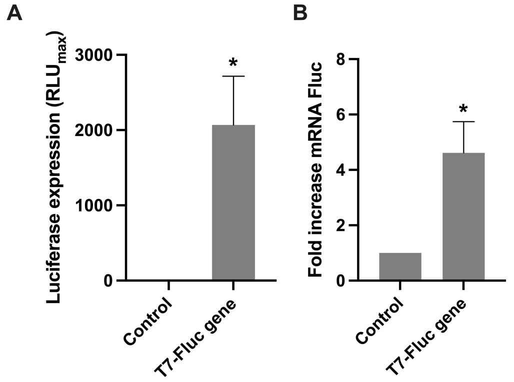

Figure S8. Comparison between mRNA (RT-qPCR) and protein levels measured by luciferase assay kit.

8. Control expression in *C. acnes* CFS acting as a biosensor

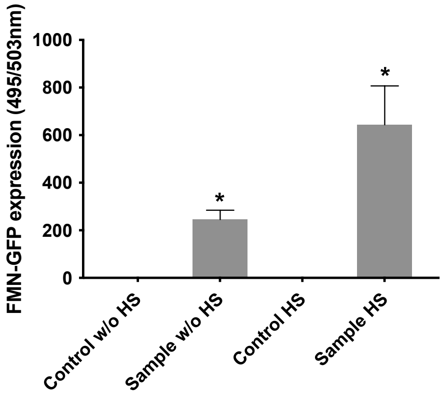

Figure S9. Luciferase expression in a *C. acnes* CFS under different temperature conditions. The T7-Fluc construct was tested for a HS stability during the CFS reaction. After 1 h at 30 °C , some reactions were incubated for 2 minutes at 42 °C for the heat shock (HS) and in parallel negative control samples were maintained at 30 °C. Results are presented as mean standard deviation (SD) of triplicate measurements ($p < 0.05$) *versus* negative control.
